# Glutamine signaling specifically activates c-Myc and Mcl-1 to facilitate cancer cell proliferation and survival

**DOI:** 10.1093/procel/pwaf029

**Published:** 2025-05-04

**Authors:** Meng Wang, Fu-Shen Guo, Dai-Sen Hou, Hui-Lu Zhang, Xiang-Tian Chen, Yan-Xin Shen, Zi-Fan Guo, Zhi-Fang Zheng, Yu-Peng Hu, Pei-Zhun Du, Chen-Ji Wang, Yan Lin, Yi-Yuan Yuan, Shi-Min Zhao, Wei Xu

**Affiliations:** Obstetrics & Gynecology Hospital of Fudan University, State Key Laboratory of Genetic and Development of Complex Phenotypes, Institutes of Biomedical Sciences, Shanghai Key Laboratory of Female Reproductive Endocrine Related Diseases and Shanghai Key Laboratory of Metabolic Remodeling, Fudan University, Shanghai 200438, China; Obstetrics & Gynecology Hospital of Fudan University, State Key Laboratory of Genetic and Development of Complex Phenotypes, Institutes of Biomedical Sciences, Shanghai Key Laboratory of Female Reproductive Endocrine Related Diseases and Shanghai Key Laboratory of Metabolic Remodeling, Fudan University, Shanghai 200438, China; Obstetrics & Gynecology Hospital of Fudan University, State Key Laboratory of Genetic and Development of Complex Phenotypes, Institutes of Biomedical Sciences, Shanghai Key Laboratory of Female Reproductive Endocrine Related Diseases and Shanghai Key Laboratory of Metabolic Remodeling, Fudan University, Shanghai 200438, China; Huashan Hospital, Fudan University, Shanghai 200040, China; Obstetrics & Gynecology Hospital of Fudan University, State Key Laboratory of Genetic and Development of Complex Phenotypes, Institutes of Biomedical Sciences, Shanghai Key Laboratory of Female Reproductive Endocrine Related Diseases and Shanghai Key Laboratory of Metabolic Remodeling, Fudan University, Shanghai 200438, China; Obstetrics & Gynecology Hospital of Fudan University, State Key Laboratory of Genetic and Development of Complex Phenotypes, Institutes of Biomedical Sciences, Shanghai Key Laboratory of Female Reproductive Endocrine Related Diseases and Shanghai Key Laboratory of Metabolic Remodeling, Fudan University, Shanghai 200438, China; Obstetrics & Gynecology Hospital of Fudan University, State Key Laboratory of Genetic and Development of Complex Phenotypes, Institutes of Biomedical Sciences, Shanghai Key Laboratory of Female Reproductive Endocrine Related Diseases and Shanghai Key Laboratory of Metabolic Remodeling, Fudan University, Shanghai 200438, China; NHC Key Laboratory of Reproduction Regulation, Shanghai Institute for Biomedical and Pharmaceutical Technologies, Shanghai 200032, China; Obstetrics & Gynecology Hospital of Fudan University, State Key Laboratory of Genetic and Development of Complex Phenotypes, Institutes of Biomedical Sciences, Shanghai Key Laboratory of Female Reproductive Endocrine Related Diseases and Shanghai Key Laboratory of Metabolic Remodeling, Fudan University, Shanghai 200438, China; Huashan Hospital, Fudan University, Shanghai 200040, China; Obstetrics & Gynecology Hospital of Fudan University, State Key Laboratory of Genetic and Development of Complex Phenotypes, Institutes of Biomedical Sciences, Shanghai Key Laboratory of Female Reproductive Endocrine Related Diseases and Shanghai Key Laboratory of Metabolic Remodeling, Fudan University, Shanghai 200438, China; Obstetrics & Gynecology Hospital of Fudan University, State Key Laboratory of Genetic and Development of Complex Phenotypes, Institutes of Biomedical Sciences, Shanghai Key Laboratory of Female Reproductive Endocrine Related Diseases and Shanghai Key Laboratory of Metabolic Remodeling, Fudan University, Shanghai 200438, China; Obstetrics & Gynecology Hospital of Fudan University, State Key Laboratory of Genetic and Development of Complex Phenotypes, Institutes of Biomedical Sciences, Shanghai Key Laboratory of Female Reproductive Endocrine Related Diseases and Shanghai Key Laboratory of Metabolic Remodeling, Fudan University, Shanghai 200438, China; Obstetrics & Gynecology Hospital of Fudan University, State Key Laboratory of Genetic and Development of Complex Phenotypes, Institutes of Biomedical Sciences, Shanghai Key Laboratory of Female Reproductive Endocrine Related Diseases and Shanghai Key Laboratory of Metabolic Remodeling, Fudan University, Shanghai 200438, China; Obstetrics & Gynecology Hospital of Fudan University, State Key Laboratory of Genetic and Development of Complex Phenotypes, Institutes of Biomedical Sciences, Shanghai Key Laboratory of Female Reproductive Endocrine Related Diseases and Shanghai Key Laboratory of Metabolic Remodeling, Fudan University, Shanghai 200438, China; Shanghai Fifth People’s Hospital, Fudan University, Shanghai 200240, China

**Keywords:** glutamine, FBW7, c-Myc, Mcl-1, glutamylation, QARS

## Abstract

Glutamine provides carbon and nitrogen to support the proliferation of cancer cells. However, the precise reason why cancer cells are particularly dependent on glutamine remains unclear. In this study, we report that glutamine modulates the tumor suppressor F-box and WD repeat domain-containing 7 (FBW7) to promote cancer cell proliferation and survival. Specifically, lysine 604 (K604) in the sixth of the 7 substrate-recruiting WD repeats of FBW7 undergoes glutaminylation (Gln-K604) by glutaminyl tRNA synthetase. Gln-K604 inhibits SCF^FBW7^-mediated degradation of c-Myc and Mcl-1, enhances glutamine utilization, and stimulates nucleotide and DNA biosynthesis through the activation of c-Myc. Additionally, Gln-K604 promotes resistance to apoptosis by activating Mcl-1. In contrast, SIRT1 deglutaminylates Gln-K604, thereby reversing its effects. Cancer cells lacking Gln-K604 exhibit overexpression of c-Myc and Mcl-1 and display resistance to chemotherapy-induced apoptosis. Silencing both *c-MYC* and *MCL-1* in these cells sensitizes them to chemotherapy. These findings indicate that the glutamine-mediated signal via Gln-K604 is a key driver of cancer progression and suggest potential strategies for targeted cancer therapies based on varying Gln-K604 status.

## Introduction

Glutamine addiction, a key metabolic hallmark of cancer cells (Wise and Thompson, 2010), refers to their heavy reliance on glutamine for proliferation and survival. This phenomenon is also observed in other proliferating cell types, such as stem cells (Vardhana et al., 2019) and activated immune cells (Carr et al., 2010). Glutamine facilitates the uptake of essential amino acids, providing nitrogen for protein and nucleotide synthesis (Ahluwalia et al., 1990; Panda et al., 2020), thus maintaining the activation of the mammalian target of rapamycin (mTOR) signaling pathway essential for cell growth (Wise and Thompson, 2010). Additionally, glutamine catabolism is promoted by the oncogenic transcription factor c-Myc (Dang et al., 1999; Gao et al., 2009), which helps glutamine inhibit apoptosis by supplying tricarboxylic acid (TCA) cycle intermediates (Le et al., 2012; Yang et al., 2014) or nitrogen (Meng et al., 2010) via glutaminase (GLS)-induced glutaminolysis (Wise et al., 2008). These mechanisms partially explain the phenomenon of glutamine addiction in cancer and proliferating cells.

Deregulation of the cell cycle is a critical factor in cancer development. Cell cycle progression is positively regulated by cyclins and cyclin-dependent kinases (CDKs) and negatively regulated by factors such as retinoblastoma protein (Rb), p53, p27, and p21. Cell cycle regulators, including cyclin D, cyclin E, c-Myc, Notch, Jun, and CDK2, are tightly controlled by the SCF (SKP1, CUL1, and F-box protein) type ubiquitin ligase complex during interphase (Ang and Harper, 2005). The F-box/WD repeat-containing protein 7 (FBW7) is an F-box subunit of the SCF-type ubiquitin ligase complex (Welcker and Clurman, 2008). FBW7 recognizes various substrates through its 7 tandem WD40 repeats, including the apoptosis inhibitor myeloid cell leukemia 1 (Mcl-1) (Inuzuka et al., 2011). Consequently, FBW7 is frequently mutated in human cancers (Jardim et al., 2014), as loss of FBW7 activates positive cell cycle regulators (Welcker and Clurman, 2008) and downregulates the expression of negative cell cycle regulators such as CDH1 (Lau et al., 2013; Pucci et al., 2000). Notably, cancers with FBW7 mutations often exhibit resistance to chemotherapy (Ye et al., 2017). FBW7 mutations are substrate-specific, meaning that different types of mutations affect FBW7 substrates in distinct ways (Welcker and Clurman, 2008).

Interestingly, apoptosis (Harley et al., 2010; Saqcena et al., 2015) and FBW7 substrates, including Mcl-1 (Wei et al., 2017), Notch (Cluntun et al., 2017), Cyclin D (Gaglio et al., 2009), Cyclin E (Gaglio et al., 2009), and Jun (Lukey et al., 2016), are all related to glutamine. However, these regulatory relationships have not been previously reported in the context of glutamine and glutamate catabolites, suggesting that the metabolites of glutaminolysis may not directly account for these effects. Moreover, glutamine deprivation impairs the growth of natural killer (NK) cells (Loftus et al., 2018), whereas inhibition of glutaminolysis does not, highlighting the signaling role of glutamine.

Amino acid signals can be generated by binding to (Cangelosi et al., 2022; Chantranupong et al., 2016) and modifying (He et al., 2018; Vo et al., 2018b) signaling proteins. Amino acid-modified proteins utilize aminoacyl tRNA synthetases (ARSs) as aminoacyl transferases (D’Hulst et al., 2020; He et al., 2018; Vo et al., 2018a). The acyl transferase activities of ARSs extend to metabolites such as lactate (Mao et al., 2024) and homocysteine (Mei et al., 2020), influencing physiological and pathological processes, including cancer development. Glutaminyl tRNA synthetase (QARS) catalyzes the lysine glutaminylation (K-Gln) of apoptosis signal-regulating kinase 1 (ASK1), thereby inhibiting apoptosis (He et al., 2018). This partly explains the survival-promoting effects of glutamine. This study aims to elucidate the mechanisms underlying glutamine addiction in cancer cells and identifies Gln-K604 as a glutamine signal that provides cancer cells with advantages in proliferation and survival.

## Results

### Glutaminylation inhibited c-Myc ubiquitination

**Figure 1.**
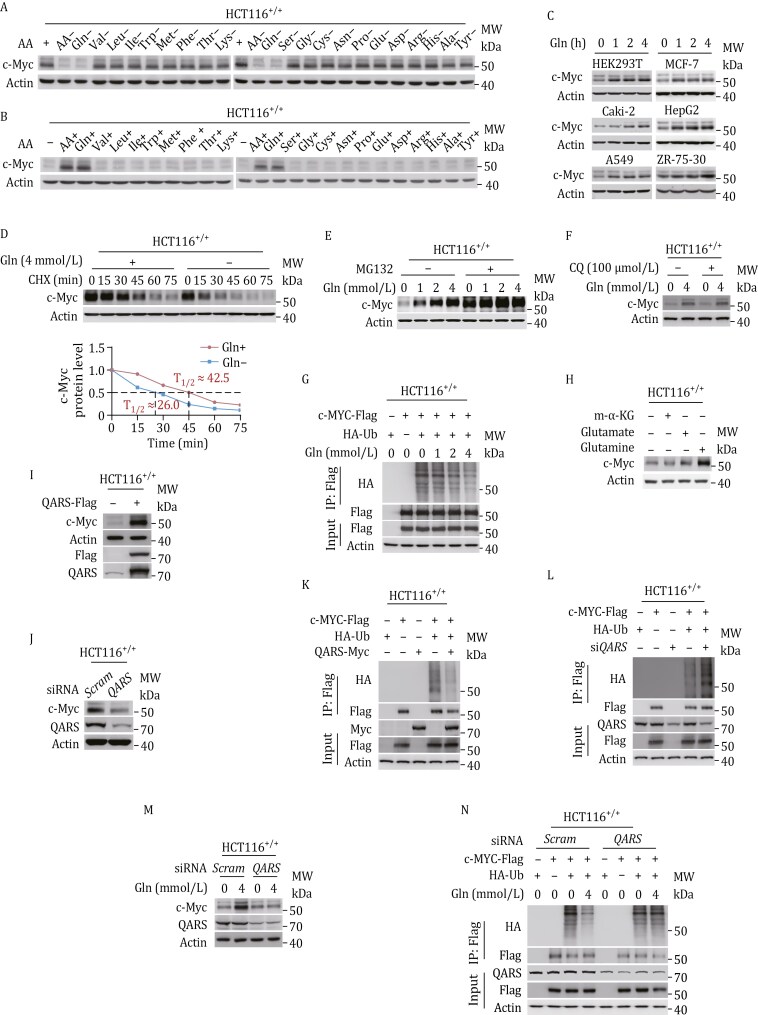
c-Myc is regulated through QARS-generated glutamine signaling. (A and B) Effects of removing (A) or adding (B) individual or all proteinogenic amino acids in RPMI 1640 medium on c-Myc protein levels in HCT116^+/+^ cells. (C) Effects of glutamine supplementation on endogenous c-Myc levels in HEK293T, Caki-2, HepG2, A549, MCF7, and ZR-75-30 cells. (D) Stability of c-Myc in the presence or absence of glutamine following cycloheximide (CHX) treatment. (E) Endogenous c-Myc levels in HCT116^+/+^ cells treated with increasing concentrations of glutamine (0, 1, 2, and 4 mmol/L) in the presence or absence of MG132. (F) Endogenous c-Myc levels in HCT116^+/+^ cells treated with 0- or 4mmol/L glutamine in the presence or absence of 100-μmol/L chloroquine. (G) Ubiquitination levels of ectopically expressed c-Myc in HCT116^+/+^ cells treated with increasing concentrations of glutamine. MG132 was used to inhibit proteasome activity (for all ubiquitination assays). (H) Effects of glutamate, α-ketoglutarate, and glutamine on c-Myc levels in HCT116^+/+^ cells. (I and J) c-Myc levels in HCT116^+/+^, QARS-overexpressing HCT116^+/+^ (I), and *QARS*-silenced HCT116^+/+^ (J) cells. (K and L) c-Myc ubiquitination levels in HCT116^+/+^, QARS-overexpressing HCT116^+/+^ (K), and *QARS*-silenced HCT116^+/+^ (L) cells. (M and N) Protein (M) and ubiquitination (N) levels of c-Myc in HCT116^+/+^ and *QARS*-silenced HCT116^+/+^ cells in the presence of 0- or 4-mmol/L glutamine.

To investigate whether glutamine reciprocally regulates c-Myc in human colon cancer HCT116^+/+^ cells, we manipulated glutamine and QARS, the substrates and enzymes responsible for glutaminylation, respectively (He et al., 2018). HCT116^+/+^ cells cultured in RPMI 1640 were deprived of either all proteogenic amino acids or glutamine alone, leading to a reduction in c-Myc levels (Fig. 1A). In contrast, supplementing amino acid-starved HCT116^+/+^ cells with either proteogenic amino acids or glutamine alone resulted in an increase in c-Myc levels (Fig. 1B). The cellular response in c-Myc levels was observed only with alterations in glutamine (Fig. 1A and 1B), suggesting that glutamine specifically regulates c-Myc expression. Furthermore, the addition of glutamine also elevated c-Myc levels in various human cell lines, including HEK293T (human embryonic kidney cells), Caki-2 (human clear cell renal cell carcinoma), HepG2 (hepatoblastoma), A549 (human lung adenocarcinoma), and MCF7 and ZR-75-30 (human breast cancer) cells (Fig. 1C).

When cycloheximide (CHX) was used to inhibit protein translation, it did not prevent the glutamine deprivation-induced decrease in c-Myc (Fig. 1D). However, inhibiting proteasomal degradation with MG132, but not lysosomal inhibition using chloroquine, resulted in c-Myc accumulation and saturated the ability of glutamine to increase c-Myc levels in HCT116^+/+^ cells (Fig. 1E and 1F). These findings, along with the observation that glutamine supplementation decreased the ubiquitination of ectopically expressed c-Myc (Fig. 1G), suggest that glutamine upregulates c-Myc expression by reducing its proteasomal degradation.

Catabolites downstream of glutamine, including glutamate and α-ketoglutarate (α-KG), did not affect c-Myc levels in HCT116^+/+^ cells (Fig. 1H), indicating that glutamine does not regulate c-Myc via its catabolic products. Overexpression of QARS increased c-Myc levels (Fig. 1I), whereas silencing QARS had the opposite effect (Fig. 1J). Moreover, QARS overexpression (Fig. 1K) and siRNA-induced *QARS* silencing (Fig. 1L) decreased and increased c-Myc ubiquitination, respectively, in HCT116^+/+^ cells. Additionally, silencing *QARS* reduced glutamine levels, which subsequently led to an increase in c-Myc protein levels (Fig. 1M) and a decrease in c-Myc ubiquitination (Fig. 1N). These results collectively suggest that glutamine signaling upregulates c-Myc expression.

### QARS glutaminylated FBW7 K604

**Figure 2.**
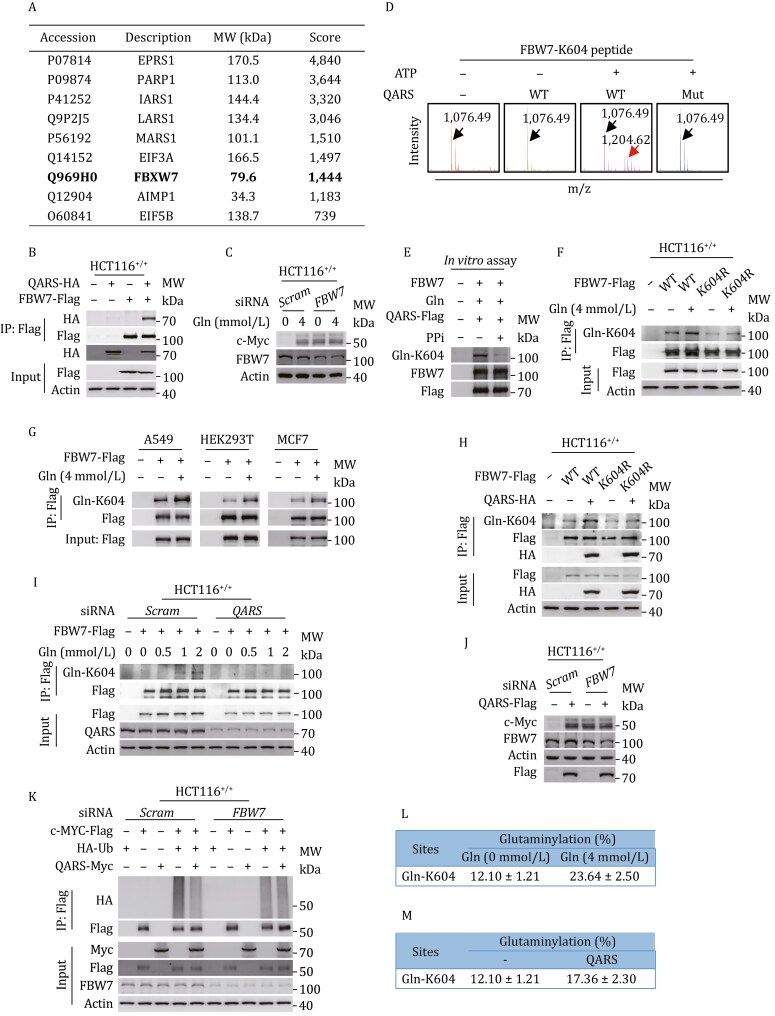
QARS glutaminylates FBW7 K604. (A and B) TAP-MS (A) and co-immunoprecipitation (B) confirm the interaction between QARS and FBW7. (C) Endogenous c-Myc levels in HCT116^+/+^ and *FBW7*-silenced HCT116^+/+^ cells cultured with 0- or 4-mmol/L glutamine. (D) *In vitro* glutaminylation assay showing the ability of recombinant QARS but not non-catalytic mutant QARS^K496A^, to modify a synthetic FBW7 peptide containing K604. The peak at m/z 1204.62 indicates the formation of glutaminylated products. The tRNA^Gln^ was added to activate the reaction in all QARS-mediated reactions. (E) QARS-mediated Gln-K604 formation in purified FBW7 in the presence or absence of pyrophosphate (PPi). (F) Gln-K604 levels in ectopically expressed FBW7^WT^ and FBW7^K604R^ mutants at 0- or 4-mmol/L glutamine in HCT116^+/+^ cells. (G) Gln-K604 levels in ectopically expressed FBW7 in A549, HEK293T, and MCF7 cells cultured with 0- or 4-mmol/L glutamine. (H) Gln-K604 levels in ectopically expressed FBW7 in HCT116^+/+^ cells with or without QARS overexpression. (I) Gln-K604 levels in ectopically expressed FBW7 in HCT116^+/+^ and *QARS*-silenced HCT116^+/+^ cells treated with increasing concentrations of glutamine (0, 0.5, 1, or 2 mmol/L). (J and K) Endogenous c-Myc levels (J) and ubiquitination levels of ectopically expressed c-Myc (K) in HCT116^+/+^ and *FBW7*-silenced HCT116^+/+^ cells with or without QARS overexpression. (L and M) The percentage of glutaminylated FBW7 in HCT116^+/+^ cells treated with 0- or 4-mmol/L glutamine (L), with or without QARS overexpression (M).

Co-immunoprecipitation experiments revealed no direct interaction between QARS and c-Myc following ectopic co-expression in HCT116^+/+^ cells (Fig. S1A), suggesting that QARS may not directly glutaminylate c-Myc. To identify QARS-interacting proteins in HCT116^+/+^ cells, we performed a co-immunoprecipitation study, which identified FBW7, a component of the SCF complex that recognizes c-Myc, as one of the top targets (Fig. 2A). This interaction was further validated through co-immunoprecipitation experiments (Fig. 2B). Silencing *FBW7* abrogated the ability of glutamine supplementation to reduce c-Myc ubiquitination and increase c-Myc protein levels (Figs. 2C and S1B), suggesting that FBW7 mediates the glutamine-induced accumulation of c-Myc.

A proteomic analysis of a tryptic FBW7 peptide library revealed glutaminylation of lysine 604 (K604) in FBW7 (Gln-K604) (Fig. S1C). A synthetic Gln-K604-containing FBW7 peptide exhibited an MS/MS spectrum identical to that of the peptide from the library (Fig. S1C), confirming the proteomic identification of Gln-K604. To verify that QARS catalyzes the formation of Gln-K604, we tested whether a synthetic K604-containing FBW7 peptide could be glutaminylated by recombinant QARS or its non-catalytic mutant *in vitro*. MS analysis showed that only recombinant QARS catalyzed the formation of Gln-K604 (Fig. 2D), and this glutaminylation was inhibited by pyrophosphate (PPi), a known inhibitor of tRNA synthetases (He et al., 2018) (Fig. 2E), further confirming that QARS glutaminylates FBW7. Using a home-made Gln-K604-specific antibody (Fig. S1D and S1E), we found that glutamine supplementation increased Gln-K604 levels in ectopically expressed wild-type FBW7 in HCT116^+/+^, HEK293T, A549, and MCF7 cells (Fig. 2F and 2G). In contrast, no increase in Gln-K604 levels was observed in the FBW7 mutant, where lysine 604 was replaced by arginine (K604R) in HCT116^+/+^ cells (Fig. 2F). Furthermore, QARS overexpression increased Gln-K604 levels in wild-type FBW7, but not in the K604R mutant (Fig. 2H), consistent with the observation that glutamine supplementation increases Gln-K604 levels in a dose-dependent manner in ectopically expressed FBW7, in a QARS-dependent manner (Fig. 2I). These results indicate that Gln-K604 is the primary glutaminylation site in FBW7 and that QARS is the main glutaminyl transferase for K604.

*FBW7* knockdown abolished the ability of QARS to elevate c-Myc levels (Fig. 2J) and decrease c-Myc ubiquitination (Fig. 2K) in HCT116^+/+^ cells, further confirming that Gln-K604 inhibits c-Myc proteasomal degradation. Additionally, MS quantification revealed that 4-mmol/L glutamine supplementation in glutamine-starved HCT116^+/+^ cells increased intracellular glutamine levels from 0.05 to 3.3 mmol/L (Fig. S1F). Glutamine supplementation also increased Gln-K604 levels from 12.10% to 23.64% (Fig. 2L). Similarly, QARS overexpression in glutamine-starved HCT116^+/+^ cells increased Gln-K604 levels from 12.10% to 17.36% (Fig. 2M). These results demonstrate that a significant fraction of Gln-K604 must be present in the cells to exert its biological effects.

### Gln-K604 specifically induced c-Myc and Mcl-1 expression

**Figure 3.**
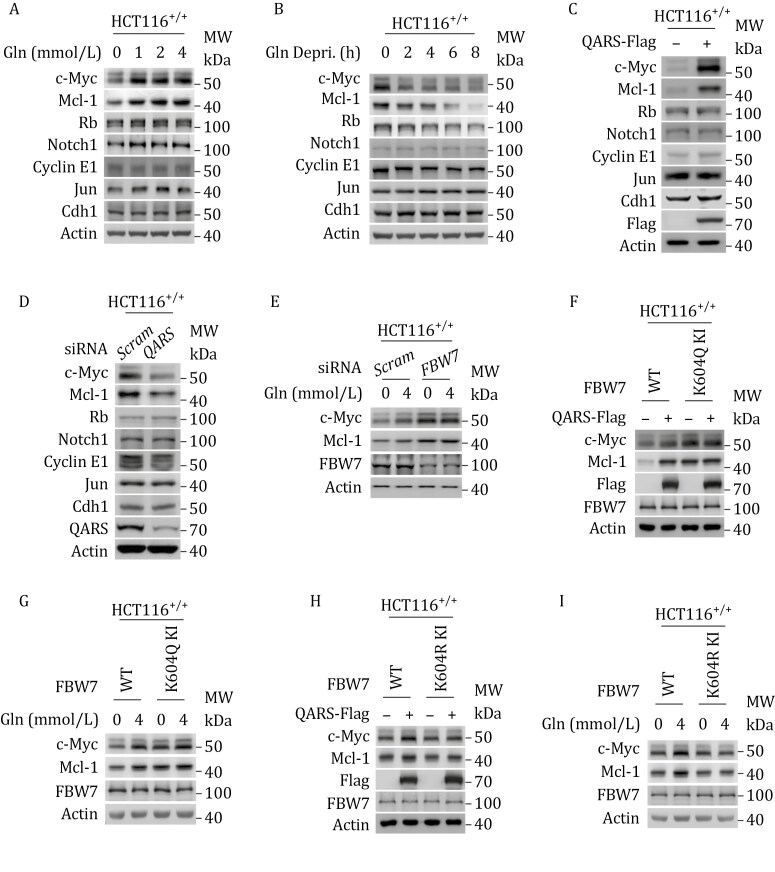
Gln-K604 specifically induces c-Myc and Mcl-1 expression. (A–D) Effects of glutamine supplementation (A) and deprivation (B), QARS overexpression (C), and *QARS* silencing (D) on endogenous c-Myc, Mcl-1, and other FBW7 substrate levels in HCT116^+/+^ cells. (E) Effects of glutamine supplementation on endogenous c-Myc and Mcl-1 levels in HCT116^+/+^ and *FBW7*-silenced HCT116^+/+^ cells. (F–I) The ability of QARS overexpression and glutamine supplementation to increase c-Myc and Mcl-1 protein levels, as compared between HCT116^+/+^ and K604Q KI HCT116^+/+^ cells (F and G) and between HCT116^+/+^ and K604R KI HCT116^+/+^ cells (H and I).

Glutamine supplementation in the culture medium increased c-Myc and Mcl-1 levels in HCT116^+/+^ cells but did not affect the levels of Rb, Notch1, Cyclin E1, c-Jun, or Cdh1 (Fig. 3A). In contrast, glutamine deprivation decreased c-Myc and Mcl-1 levels, with no effect on the other FBW7 substrates (Fig. 3B). These results suggest that glutamine specifically regulates c-Myc and Mcl-1 expression.

Overexpression of QARS (Fig. 3C) and *QARS* silencing (Fig. 3D) mimicked the effects of glutamine supplementation and deprivation on c-Myc and Mcl-1 levels, indicating that glutaminylation specifically regulates the expression of these 2 proteins. Furthermore, siRNA-mediated silencing of *FBW7* abrogated the ability of glutamine supplementation (Fig. 3E) to upregulate c-Myc and Mcl-1 levels in HCT116^+/+^ cells, which was consistent with the observation that *FBW7* silencing inhibited the ability of QARS to increase c-Myc levels (see Fig. 2J). This supports the conclusion that Gln-K604 specifically regulates c-Myc and Mcl-1 expression. Additionally, the levels of Mcl-1 were not altered by the administration of glutamate or α-ketoglutarate (α-KG) in HCT116^+/+^ cells (Fig. S2A).

We also examined other cell cycle regulators, including Cyclin A, Cyclin B, and Cyclin D, and found that, such as Cyclin E, these regulators were unaffected by glutamine supplementation (Fig. S2B). To further confirm the role of Gln-K604 in regulating c-Myc and Mcl-1, we generated a cell line with a K604Q mutation in FBW7 (FBW7^K604Q^) in the HCT116^+/+^ genome (K604Q-KI), which mimics constitutive glutaminylation of FBW7 (Fig. S2C). This mutation resulted in elevated c-Myc and Mcl-1 levels (Fig. 3F and 3G). In contrast, replacing K604 with arginine (FBW7^K604R^) in the HCT116^+/+^ genome (K604R-KI) to mimic a constitutive non-glutaminylated FBW7 (Fig. S2C) resulted in persistently lower levels of c-Myc and Mcl-1 (Fig. 3H and 3I). Notably, neither glutamine supplementation nor QARS overexpression affected c-Myc or Mcl-1 expression in the K604Q-KI or K604R-KI HCT116^+/+^ cells (Fig. 3F–I). Finally, the K604Q-KI and K604R-KI mutations had minimal impact on the other FBW7 substrates tested (Fig. S2D). These findings confirm that Gln-K604 specifically induces the accumulation of c-Myc and Mcl-1.

### Gln-K604 disrupts the binding of c-Myc and Mcl-1 to FBW7

**Figure 4.**
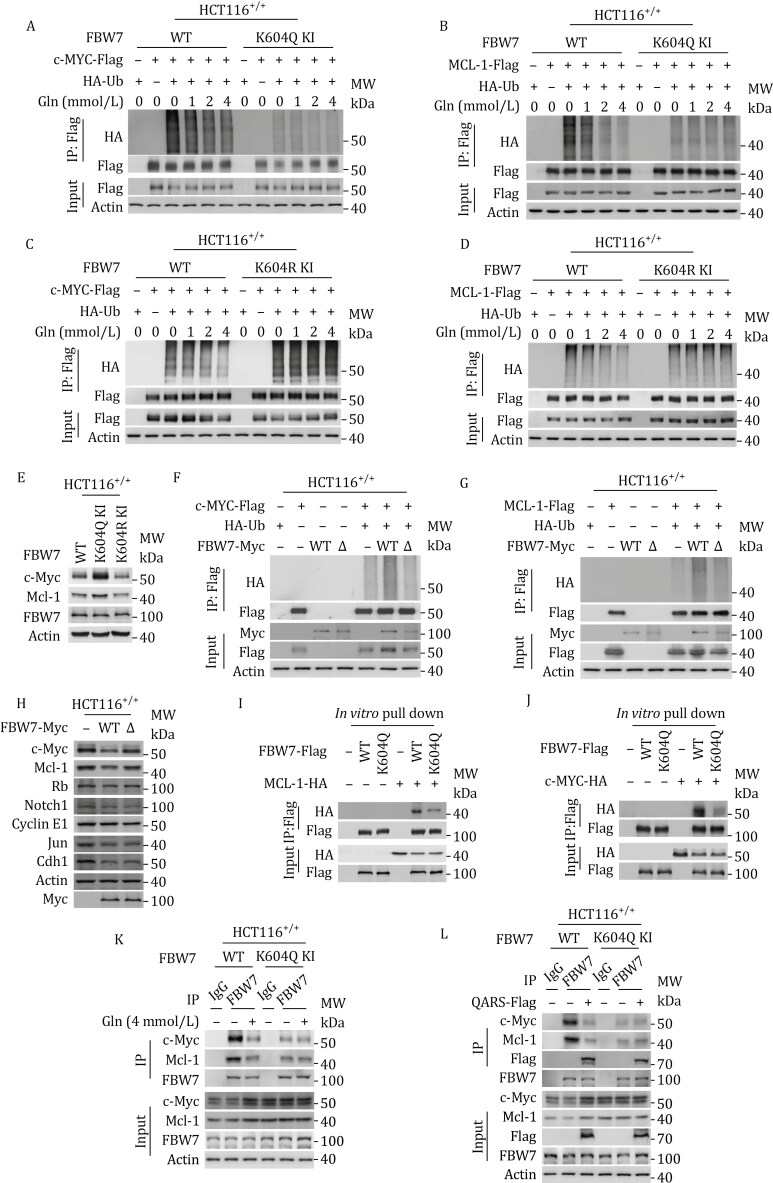
Gln-K604 disrupts the binding of c-Myc and Mcl-1 to FBW7. (A–D) Ubiquitination levels of ectopically expressed c-Myc and Mcl-1 in response to glutamine supplementation between HCT116^+/+^ and K604Q KI cells (A and B) and between HCT116^+/+^ and K604R KI cells (C and D). (E) Endogenous c-Myc and Mcl-1 levels in HCT116^+/+^, K604Q KI, and K604R KI cells. (F and G) Ubiquitination levels of ectopically expressed c-Myc (F) and Mcl-1 (G) in HCT116^+/+^, FBW7-overexpressing HCT116^+/+^, and FBW7Δ^6thWD40^-overexpressing HCT116^+/+^ cells. (H) Endogenous c-Myc and Mcl-1 levels in HCT116^+/+^, FBW7-overexpressing HCT116^+/+^, and FBW7Δ^6thWD40^-overexpressing HCT116^+/+^ cells. (I and J) The ability of recombinant FBW7 and FBW7 K604Q mutants to pull down Mcl-1 (I) and c-Myc (J) *in vitro*. (K and L) The binding of c-Myc and Mcl-1 to FBW7 and K604Q at 0 mmol/L and 4 mmol/L glutamine (K), with or without QARS overexpression (L) in HCT116^+/+^ and K604Q KI cells.

K604Q-KI (Fig. 4A and 4B) and K604R-KI (Fig. 4C and 4D) mutations resulted in decreased and increased ubiquitination levels of c-Myc and Mcl-1, respectively, which was consistent with the corresponding changes in the endogenous levels of these proteins in K604Q-KI and K604R-KI cells (Fig. 4E). However, glutamine supplementation failed to reduce the ubiquitination of c-Myc and Mcl-1 in both K604Q-KI and K604R-KI HCT116^+/+^ cells (Fig. 4A–D), and no alterations were observed in the ubiquitination levels of Cyclin E1, Notch1, or c-Jun (Fig. S3A–C). These results suggest that Gln-K604 specifically inhibits the degradation of c-Myc and Mcl-1.

K604 is localized to the sixth WD40 domain of FBW7, which is involved in substrate recognition (Fig. S3D). This suggests that c-Myc and Mcl-1 may be recruited to the sixth WD40 domain for degradation. Deletion of the sixth WD40 domain in FBW7 (Δ6thWD40, Fig. S3D) led to increased ubiquitination of Rb, Cyclin E1, Notch1, Jun, and Cdh1 but had minimal effects on the ubiquitination and expression of c-Myc and Mcl-1 (Figs. 4F–H and S3E–G). These findings confirm that the sixth WD40 domain of FBW7 specifically recruits c-Myc and Mcl-1 for degradation.

The recombinant Gln-K604 mimetic, K604Q, exhibited a weaker ability to pull down both c-Myc and Mcl-1 *in vitro* (Fig. 4I and 4J). Co-immunoprecipitation analysis revealed that the interaction between FBW7 and c-Myc and Mcl-1 progressively diminished in a glutamine dose-dependent manner (Fig. S3H). Structural analysis of the interaction between FBW7^WT^ and FBW7^Gln-K604^ with c-Myc and Mcl-1 revealed that Gln-K604 significantly altered the binding pattern of these proteins to FBW7 (Fig. S3I). This suggests that Gln-K604 disrupts the binding of c-Myc and Mcl-1 to FBW7. This conclusion is supported by the observation that both glutamine supplementation and QARS overexpression (Figs. 4K, 4L, S3J and S3K) decreased the binding of c-Myc and Mcl-1 to FBW7 in HCT116^+/+^ cells. Moreover, the co-immunoprecipitation of c-Myc and Mcl-1 with FBW7 in K604Q-KI and K604R-KI cells was unaffected by either glutamine supplementation or QARS overexpression (Figs. 4K, 4L, S3J and S3K). These results confirm that Gln-K604, produced by glutamine and QARS, specifically disrupts the binding of c-Myc and Mcl-1 to FBW7, preventing their degradation.

### Gln-K604 drives glutamine uptake, anabolism, and cell proliferation

**Figure 5.**
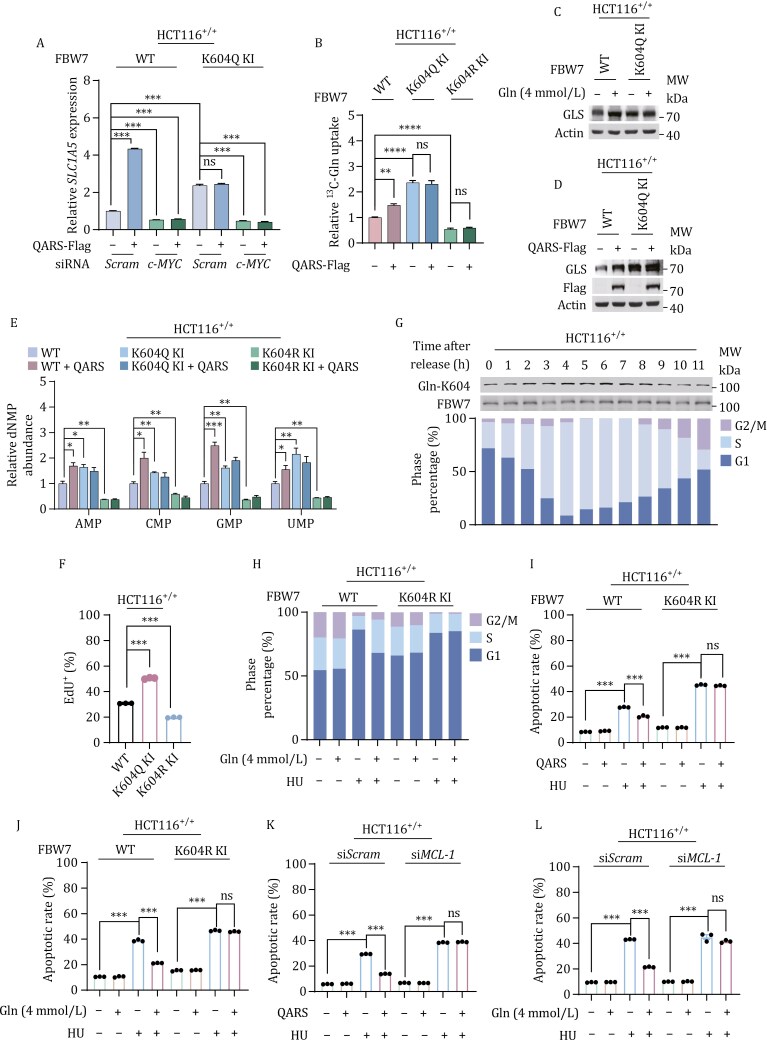
Cell cycle-dependent Gln-K604 promotes anabolism and apoptosis resistance. (A) Comparison of the mRNA levels of *SLC1A5* in HCT116^+/+^ and K604Q KI cells with or without QARS overexpression, in the presence and absence of *c-MYC*-silencing (*n* = 4). (B) Relative cellular ^13^C-glutamine levels in HCT116^+/+^, K604Q KI, and K604R KI cells, as well as in QARS-overexpressing cells. Following metabolic labeling, cells were chased with ^13^C-glutamine for 15 min. Data are presented as the mean ± SEM. *P < 0.05, **P < 0.01, ***P < 0.001. (C and D) Effects of glutamine supplementation (C) and QARS overexpression (D) on GLS protein levels in HCT116^+/+^ and K604Q KI cells. (E) Intracellular AMP, GMP, CMP, and UMP levels in HCT116^+/+^, K604Q KI, and K604R KI cells, as detected through LC-MS in the presence or absence of QARS overexpression. (F) EdU assay for DNA synthesis in HCT116^+/+^, K604Q KI, and K604R KI cells. (G) Gln-K604 levels and cell cycle distribution in HCT116^+/+^ cells synchronized at the G1/S phase using DTB, assessed by immunoblotting and FCM, respectively. (H) Cell cycle distribution in untreated and HU-arrested HCT116^+/+^ and K604R KI cells with or without 4-mmol/L glutamine, detected by FCM (*n* = 3). (I and J) Apoptotic rates of untreated and HU-treated HCT116^+/+^ and K604R KI cells, assessed by FCM (*n* = 3). (I) Effects of QARS overexpression. (J) Effects of glutamine supplementation. (K and L) Apoptotic rates of untreated and HU-treated HCT116^+/+^ and *MCL-1*-silenced HCT116^+/+^ cells, assessed by FCM (*n* = 3). (K) Effects of QARS overexpression. (L) Effects of glutamine supplementation.

K604Q-KI and QARS overexpression in HCT116^+/+^ cells significantly elevated the mRNA levels of SLC1A5, the primary glutamine transporter, in a c-Myc-dependent manner (Fig. 5A). Similarly, K604Q-KI and K604R-KI cells exhibited higher and lower ^13^C-glutamine uptake abilities, respectively, and QARS overexpression promoted ^13^C-glutamine uptake only in HCT116^+/+^ cells, but not in K604Q-KI or K604R-KI cells (Fig. 5B). These findings confirm that Gln-K604 enhances glutamine uptake. Moreover, K604Q-KI cells consistently exhibited increased glutamine catabolic GLS levels, independent of glutamine supplementation (Fig. 5C) or QARS overexpression (Fig. 5D), suggesting that both glutamine uptake and catabolism, which are activated by c-Myc (Gao et al., 2009; Panda et al., 2020), are promoted by Gln-K604.

In line with the increase in nucleotide synthesis precursors, QARS overexpression elevated nucleotide levels in HCT116^+/+^ cells, but not in K604Q-KI or K604R-KI cells. K604Q-KI and K604R-KI cells showed higher and lower nucleotide levels, respectively, compared with HCT116^+/+^ cells (Fig. 5E). Additionally, these cells exhibited higher and lower EdU incorporation into chromosomes, respectively (Figs. 5F and S4A). Release assays from DTB-synchronized HCT116^+/+^ cells revealed elevated Gln-K604 levels during late G1 and throughout the S phase (Fig. 5G). These results suggest that Gln-K604 promotes anabolic processes and cell proliferation (Kondo et al., 2000). This hypothesis was further supported by the use of hydroxyurea (HU) or alisertib (ALS) to induce G1/S and G2/M phase arrest (Apraiz et al., 2017; Ding et al., 2020; Wang et al., 2015; Winnicki et al., 2013), respectively. Glutamine supplementation alleviated these cell cycle arrests in HCT116^+/+^ cells but not in K604R-KI cells (Figs. 5H and S4B).

### Gln-K604 prevents cell cycle arrest-induced apoptosis

QARS overexpression or glutamine supplementation inhibited both HU-induced (Fig. 5I and 5J) and ALS-induced apoptosis (Fig. S4C and S4D) in HCT116^+/+^ cells, whereas *QARS* silencing had the opposite effect (Fig. S4E and S4F). However, these apoptosis-inhibiting effects of QARS and glutamine supplementation were not observed in K604R-KI cells (Figs. 5I, 5J, S4C and S4D). Additionally, in glutamine-starved cells, which had low Mcl-1 (see Fig. 3B), Gln-K604 upregulated the expression of the anti-apoptotic protein Mcl-1. *MCL-1* knockdown in HCT116^+/+^ cells partially abrogated the protective effects of QARS overexpression or glutamine supplementation against HU- (Fig. 5K and 5L) or ALS-induced apoptosis (Fig. S4G and S4H). These results suggest that Gln-K604 partially inhibits apoptosis through the upregulation of Mcl-1 expression.

### SIRT1 eliminates Gln-K604 and its effects

**Figure 6.**
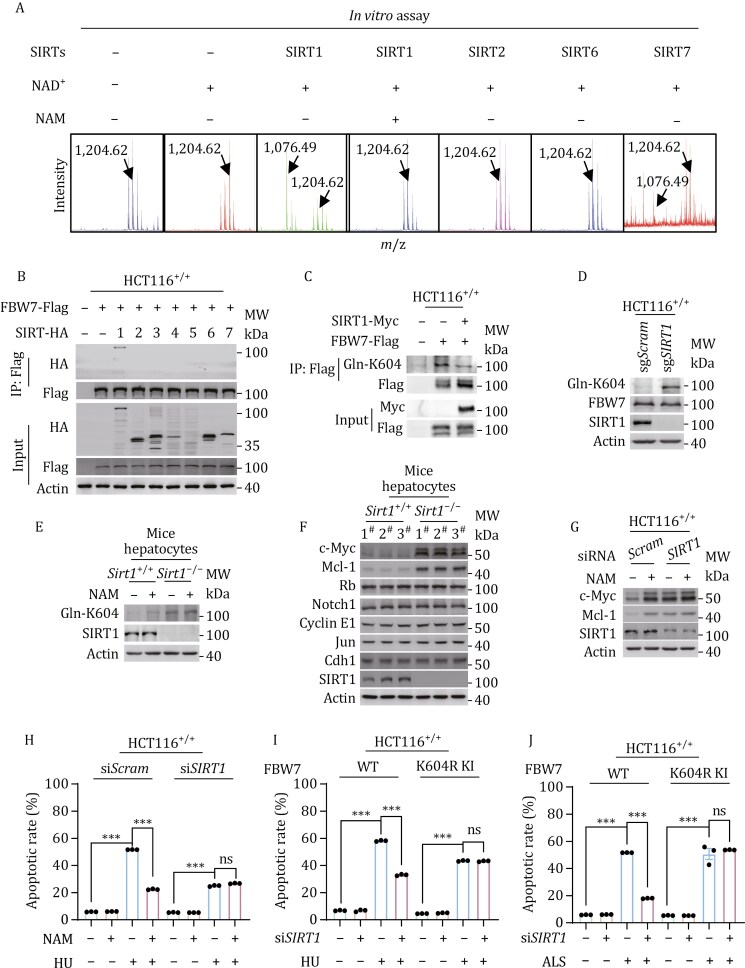
SIRT1 removed Gln-K604 and reversed Gln-K604 effects ***in vitro.*** (A) MS analysis for the ability of recombinant SIRT1, SIRT2, SIRT6, and SIRT7 to remove Gln-K604 from a synthetic Gln-K604-containing peptide. (B) Co-immunoprecipitation analysis of interactions between FBW7 and SIRTs in HCT116^+/+^ cells. (C) Gln-K604 levels in HCT116^+/+^ cells ectopically expressed FBW7, with or without SIRT1 co-expression. (D) Gln-K604 levels in HCT116^+/+^ and *SIRT1* knockout HCT116^+/+^ cells. (E) Gln-K604 levels in hepatocytes from *Sirt1*^*+/+*^ and *Sirt1*^*−/−*^ C57 mice, with or without 5-mmol/L NAM treatment. (F) Levels of FBW7 substrate protein in hepatocytes from *Sirt1*^*+/+*^ and *Sirt1*^*−/−*^ C57 mice. (G) c-Myc and Mcl-1 levels in HCT116^+/+^ and *SIRT1-*silenced HCT116^+/+^ cells with or without 5-mmol/L NAM treatment. (H) Apoptotic rates of HCT116^+/+^ and *SIRT1*-silenced HCT116^+/+^ cells with and without HU-induced apoptosis, as detected by FCM (*n* = 3). (I and J) Apoptotic rates of HCT116^+/+^, *SIRT1*-silenced HCT116^+/+^, K604R KI, *SIRT1*-silenced K604R KI cells with and without HU- (I) and ALS-induced (J) apoptosis, detected by FCM (*n* = 3).

We screened for potential deglutaminylases of Gln-K604 among sirtuin family deacetylases with amidase activity (He et al., 2018). SIRT1 was found to remove Gln-K604 in an NAD^+^-dependent and nicotinamide (NAM)-inhibitable manner in the synthetic FBW7 peptide (Fig. 6A), confirming its *in vitro* deglutaminylase activity. Of the SIRTs tested, only SIRT1 interacted with FBW7 when ectopically co-expressed in HCT116^+/+^ cells (Fig. 6B), suggesting that SIRT1 acts directly on FBW7. Furthermore, SIRT1 overexpression decreased Gln-K604 levels (Fig. 6C), while *SIRT1* knockout increased endogenous Gln-K604 levels (Fig. 6D), indicating that SIRT1 possesses deglutaminylase activity in cells.

The *in vivo* deglutaminylase activity of SIRT1 was confirmed using a *Sirt1* knockout C57BL/6 mouse model (*Sirt1*^*−/−*^). Hepatocytes from *Sirt1*^*−/−*^ mice exhibited higher Gln-K604 levels compared with wild-type hepatocytes. Additionally, *Sirt1* knockout nullified the ability of NAM to elevate Gln-K604 (Fig. 6E). Hepatocytes from *Sirt1*^*−/−*^ mice also showed elevated c-Myc and Mcl-1 levels, while the levels of other FBW7 substrates remained unchanged (Fig. 6F). These findings confirm that SIRT1 acts as a deglutaminylase of Gln-K604 and regulates c-Myc and Mcl-1 through this modification.

*SIRT1* knockdown in HCT116^+/+^ cells abrogated the NAM-induced increase in c-Myc and Mcl-1 levels (Fig. 6G), resulting in resistance to HU-induced apoptosis (Fig. 6H). Additionally, *SIRT1* silencing phenocopied the effects of glutamine, rescuing cell cycle progression from HU- or ALS-induced cell cycle arrest (Fig. S5A and S5B). *SIRT1* silencing also desensitized cells to apoptosis induced by HU (Fig. 6I) or ALS (Fig. 6J). These results confirm that SIRT1 inhibits the ability of Gln-K604 to prevent apoptosis.

### Precise chemotherapy-sensitizing strategies for Gln-K604-intact and Gln-K604-null cancers

**Figure 7.**
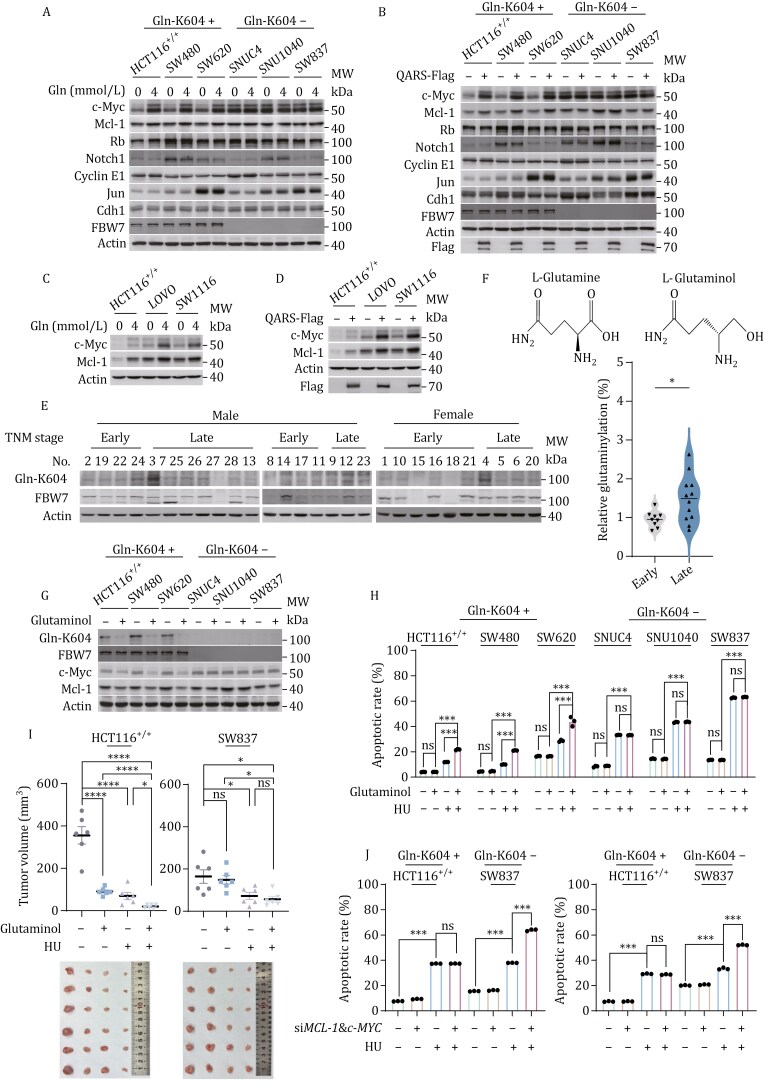
Precise chemotherapy-sensitizing approaches for Gln-K604-intact and Gln-K604-null cancers. (A and B) Levels of c-Myc, Mcl-1, and other FBW7 substrates in Gln-K604-intact (HCT116^+/+^, SW480, and SW620) and Gln-K604-null (SNUC4, SNU1040, and SW837) colorectal cancer cells, with changes upon 4-mmol/L glutamine supplementation (A) or QARS overexpression (B). (C and D) c-Myc and Mcl-1 levels in HCT116^+/+^ and FBW7 point mutations (LOVO and SW1116) in Gln-K604-intact cells, with changes upon 4-mmol/L glutamine supplementation (C) or QARS overexpression (D). (E) Western blot analysis of Gln-K 604 levels in paired early- and late-stage colorectal cancer tissue samples (*n* = 14 pairs). (F) Structure comparison of L-glutaminol and L-glutamine. (G and H) Gln-K604, c-Myc, and Mcl-1 levels (G) and apoptosis rates (H) in Gln-K604-intact and Gln-K604-null cancer cells following glutaminol treatment and/or HU treatment, detected by Western blot and FCM (*n* = 3). Cells are cultured in 4-mmol/L glutamine. (I) Tumor growth in xenograft models (HCT116^+/+^ and SW837), with or without HU and/or glutaminol treatment (*n* = 6). (J) Apoptotic rates of HCT116^+/+^, SW837, *MCL-1/c-MYC*-silenced HCT116^+/+^, and *MCL-1/c-MYC*-silenced SW837 cells with or without HU or ALS treatment, under glutamine depletion (0-mmol/L glutamine), detected by FCM (*n* = 3).

FBW7 point mutations and truncations are frequently observed in colorectal and other cancers, with most FBW7 truncations lacking the WD domain (Fig. S6A). We compared the apoptotic responses of colorectal cancer cell lines with intact Gln-K604 (Gln-K604^+^; HCT116^+/+^, SW480, SW620) and those null Gln-K604 (Gln-K604-; SNUC4, SNU1040, SW837). In Gln-K604-null cells, c-Myc and Mcl-1 levels remained high and were insensitive to variations in glutamine supplementation, QARS overexpression, or *QARS* silencing. In contrast, glutamine supplementation and starvation, along with QARS overexpression and silencing, altered c-Myc and Mcl-1 levels in Gln-K604-intact cells (Figs. 7A, 7B, S6B and S6C). Similarly, c-Myc and Mcl-1 responses to glutamine and QARS were observed in LOVO and SW1116 colorectal cancer cells harboring Gln-K604-intact FBW7 point mutations (Fig. 7C and 7D), suggesting that the loss of Gln-K604 contributes to the upregulation of c-Myc and Mcl-1 expression.

We also collected pathological samples from 14 pairs of early-stage (TNM stage 1/2) and late-stage (TNM stage 3/4) colorectal cancer patients. FBW7 was absent in 3 of the samples, although no correlation was found between FBW7 absence and the degree of malignancy. When we compared the levels of Gln-K604 in these samples, we found that late-stage samples had significantly higher levels of Gln-K604 compared with early-stage samples (Fig. 7E).

To further confirm that the loss of Gln-K604 promotes c-Myc and Mcl-1 overexpression and apoptosis resistance, we evaluated the effects of glutaminol, a structural analog of glutamine (Fig. 7F), in cancer cells. Glutaminol decreased Gln-K604, c-Myc, and Mcl-1 levels in Gln-K604-intact colorectal cancer cells but had no effect in Gln-K604-null cells (Fig. 7G), suggesting that glutaminol inhibits glutamine signaling. Moreover, glutaminol treatment increased apoptosis rates in Gln-K604-intact cells but not in Gln-K604-null cells. Glutaminol also potentiated HU- (Fig. 7H) and ALS- (Fig. S6D) induced apoptosis only in Gln-K604-intact colorectal cancer cells. These findings were further supported by mouse xenograft models, where glutaminol inhibited the growth of Gln-K604-intact HCT116^+/+^ xenografts but not Gln-K604-null SW837 xenografts, although both xenografts were inhibited by HU (Fig. 7I).

Finally, we hypothesized that inactivation of c-Myc and Mcl-1 would sensitize Gln-K604-null cancers to chemotherapy. Indeed, simultaneous silencing of *c-MYC* and *MCL-1* sensitized SW837 cells to HU- and ALS-induced apoptosis but had no effect in HCT116^+/+^ cells (Fig. 7J).

## Discussion

This study reveals that glutamine promotes anabolism and apoptosis resistance, key factors for cell proliferation, through signaling mechanisms. The modification of K604 in the cell cycle suppressor FBW7 specifically prevents FBW7 from degrading c-Myc and Mcl-1, while leaving other FBW7 substrates such as Rb, Notch1, Cyclin E1, Jun, and Cdh1 unaffected. This is due to the location of K604 in the sixth WD domain of FBW7, which recognizes c-Myc and Mcl-1. The specific regulation of c-Myc and Mcl-1 by K604 is further confirmed by the observation that in cells harboring the K604Q-KI mutation, glutamine’s regulatory effect on the ubiquitination levels of c-Myc and Mcl-1 is abolished. Furthermore, c-Myc enhances glutamine uptake and catabolism, promoting anabolic processes such as nucleotide and DNA synthesis, which creates a positive feedback loop for glutamine signaling. Mcl-1, in turn, prevents apoptosis, which is often triggered by cell cycle arrest—a common event in proliferating cells.

SIRT1, an NAD^+^-dependent deacetylase, removes Gln-K604 and is activated by low-energy conditions that inhibit anabolism. The resistance of SIRT1 to both proliferation and apoptosis is consistent with its role in inducing G_1_ phase arrest (Zhou et al., 2015) and its reduced activity in certain cancers (Deng, 2009). Additionally, SIRT1 activation through resveratrol decreases cell viability and increases apoptosis in a dose-dependent manner (Liu et al., 2018), whereas SIRT1 inhibition suppresses shikonin-induced apoptosis (Jeung et al., 2016). The association between increased SIRT1 expression and improved prognosis in various cancers (Jung et al., 2013; Noguchi et al., 2013) further supports the role of Gln-K604 as a central metabolic signal for proliferation (Fig. S7).

Cells utilize Gln-K604 as a signal promoting proliferation, likely because glutamine serves as a source of carbon, energy, and nitrogen for *de novo* nucleotide and DNA synthesis (Thompson, 2011; Yoo et al., 2020). Therefore, glutamine is a key marker of proliferation. Cellular glutamine levels are positively correlated with c-Myc levels (Loftus et al., 2018). Glutamine deprivation induces cell cycle arrest in both non-transformed primary cells and K-Ras mutant cancer cells (Saqcena et al., 2015). In the tumor microenvironment, glutamine is highly synthesized, allowing cancer cells to maintain sufficient levels (Yang et al., 2016). These findings align with the present study, where Gln-K604 activates c-Myc to produce glutamine and also activates Mcl-1 to prevent apoptosis during proliferation. The enzyme responsible for Gln-K604 formation, QARS, binds to ASK1 and suppresses its pro-apoptotic activity (Ko et al., 2001), providing another mechanism for apoptosis inhibition.

Our results suggest that the inability of cells with FBW7 point mutations and truncations to suppress c-Myc and Mcl-1 may contribute to chemotherapy resistance in cancers lacking the sixth WD domain (Ye et al., 2017). This insight could lead to the development of precise chemotherapeutic strategies tailored to cancers with different Gln-K604 statuses. For example, K604-Gln-intact cancers could be sensitized to HU and ALS treatments through the Gln-K604 inhibitor glutaminol, whereas Gln-K604-null cancers could be sensitized by silencing *c-MYC* and *MCL-1*. Further clinical investigation is needed to validate these findings in clinical settings, including at the cellular and xenograft levels.

## Methods and Materials

### Cells

HCT116^+/+^ and Caki-2 cells were cultured in Roswell Park Memorial Institute (RPMI) 1640 (Boster, China) supplemented with 10% fetal bovine serum (FBS) (Gibco, Carlsbad, USA), 100-units/mL penicillin (Invitrogen, Carlsbad, USA), and 100-mg/mL streptomycin (Invitrogen, Carlsbad, USA) at 37°C in humidified 5% CO_2_ incubator.

HEK293T, A549, MCF7, ZR-75-30, SW480, SW620, SW837, SNU1040, LOVO, SW1116, and SNUC4 cells were cultured in Dulbecco’s Modified Eagle’s Medium (DMEM) (Gibco) supplemented with 10% FBS, penicillin, and streptomycin at 37°C in humidified 5% CO_2_ incubator.

For amino acids deprivation studies, cells were incubated in serum-free RPMI 1640 (Boster) or DMEM lacking individual amino acids for 2 h. Subsequent amino acid supplementation experiments were performed by adding specific amino acids to the deprivation media for 1 h.

For cycloheximide treatment, cells were exposed to CHX for 15–75 min prior to harvest.

For MG132 treatment, cells were exposed to 10-μmol/L MG132 for 6 h preceding harvest.

For nicotinamide (NAM) treatment, 5-mmol/L NAM was administered to cells 6-h preharvest.

For L-glutaminol treatment, following 2-h serum starvation, cells were treated with 4-mmol/L L-glutaminol for 1 h.

### Animal models

Generation of *Sirt1* conditional knock-out mice was described previously. Five to six weeks male BALB/c nude mice were subcutaneously injected with 2 × 10^6^ cancer cells resuspended in 100-μL PBS. For L-glutaminol treatment, L-glutaminol was dissolved in 2.5% ethanol in PBS (*v*/*v*) and administered daily via oral gavage (1 mg/kg in 100-μL vehicle) from days 8 to 22. For HU treatment, HU was dissolved in 0.9% saline and delivered by intraperitoneal injection (100 mg/kg) every other day.

Tumor volume was calculated according to the equation: Volume (mm^3^) = (length × width^2^)/2. Mice were sacrificed before tumor reached 1.5 cm in any dimension.

### Western blot

Cultured cells were lysed in ice-cold lysis buffer (50 mmol/L Tris-HCl pH 7.5, 150 mmol/L NaCl, 0.5% Nonidet P-40, aprotinin, leupeptin, pepstatin, Phenylmethanesulfonyl fluoride) for 30 min at 4°C. Lysates were centrifuged at 12,000 rpm for 15 min at 4°C. Supernatants were collected and boiled with 5× SDS loading buffer. Equal amounts of cell lysates were subjected to SDS-PAGE. Proteins were transferred to nitrocellulose membranes (Cytiva) and blocked with 5% fat-free milk (BD Biosciences) before immunoblotting with primary antibodies. Signals were detected using Typhoon FLA 9500 (GE, USA).

Antibodies against FBW7 rabbit (#A301-720A, 1:5,000, RRID: AB_1210897) was purchased from BETHYL. Antibodies against c-Myc (Cat# ab32072, 1:3,000, RRID: AB_731658) and Rb (Cat# ab181616, 1:1000, RRID: AB_2848193) were purchased from Abcam. Antibodies against Mcl-1 (Cat# 94296S, 1:2,000, RRID: AB_2722740), Notch1 (Cat# 3608S, 1:1000, RRID: AB_2153354), Cyclin E1 (Cat# 20808S, 1:1000, RRID: AB_2783554), GLS (Cat# 56750S, 1:1000), c-Jun (Cat# 9165S, 1:1000, RRID: AB_2130165), and SIRT1 (Cat# 9475S, 1:1000, RRID: AB_2617130) were purchased from Cell Signaling Technology. Antibodies against QARS (Cat# 12645-1-AP, 1:1000, RRID: AB_2098676) was purchased from Proteintech. Antibodies against FZR1 (Cat# GTX111200, 1:1000, RRID: AB_11173217) were purchased from GeneTex. Antibodies against Flag (Cat# M20008, RRID: AB_2713960), Myc (Cat# M20002, RRID: AB_2861172), and HA (Cat# M20003, RRID: AB_2864345) were purchased from Abmart. Goat anti-rabbit IgG (Cat# 111-035-003, RRID: AB_2313567) and Goat anti-mouse IgG (Cat# 115-035-003, RRID: AB_10015289) were purchased from Jackson.

For generation of Anti-Gln-K604 (1:250) antibody, synthetic peptide (DNILVSGNADSTVK604GlnIWDIK) was conjugated to the carrier protein keyhole limpet hemocyanin as antigen. Rabbits were immunized with the conjugate by subcutaneous injection every 2 weeks for 4 times before they were sacrificed. Then, collected blood and centrifuged to harvest serum, followed by purification with antigen peptide. The specificity was verified by blot assay and Western blot.

### Flow cytometry

For apoptosis analysis, cells treated with hydroxyurea (HU) (Sigma Aldrich) or Alisertib (ALS) (MedChemExpress, New Jersey, USA) for 48 h were digested with non-EDTA trypsin and stained using Annexin V-Alexa Fluor488/PI Apoptosis Detection Kit (#40305ES, YEASEN, Shanghai, China). Apoptotic populations were quantified using FACSCalibur Flow Cytometry (BD Biosciences).

For cell cycle analysis, cells treated with HU or ALS for 12 h were digested with trypsin, fixed in 70% ethanol at 4°C overnight, and stained with PI (Sigma Aldrich) for 15 min at room temperature. Cell cycle distribution was analyzed using FACSCalibur Flow Cytometry (BD Biosciences).

### RNA isolation and reverse transcription quantitative PCR

Total cellular RNA was extracted with TRIzol reagent (TransGen Biotech), and 1-μg RNA was reverse-transcribed with HiScript II 1st Strand cDNA Synthesis Kit (+ gDNA wiper) (#R212, Vazyme). Quantitative PCR was performed with ChamQ SYBR qPCR Master Mix (#Q311, Vazyme) on CFX Connect Real-Time PCR System (Bio-Rad) and analyzed relative mRNA expressions. The specific primers used for qRT-PCR are listed in Table S1.

### Plasmids constructs and transfection

Whole length human FBW7, QARS, c-MYC, MCL-1, NOTCH1, CCNE1, and JUN were amplified from HCT116^+/+^ cDNA and cloned into the Xho I and EcoR I restriction sites of the pcDNA3.1-Flag/HA/Myc vector using CloneExpress multiS One Step Cloning Kit (#C113-02, Vazyme, Nanjing, China). Site-directed mutagenesis of FBW7 was performed with the Mut Express MultiS Fast Mutagenesis kit (#C215-01, Vazyme). Plasmid transfections were carried out using Lipo8000 Transfection Reagent (Beyotime Biotechnology, China) according to the manufacturer’s instructions. Primer sequences are listed in Table S1.

### Small RNA interference

Synthetic siRNAs targeting *FBW7/QARS/c-MYC/MCL-1* and scrambled negative control siRNA were transfected using Lipo8000 Transfection Reagent (Beyotime Biotechnology) according to the manufacturer’s protocol. Knockdown efficiency was verified by Western blot. Primer sequences are listed in Table S1.

### CRISPR/Cas9 genomic knock-in

FBW7 K604Q and FBW7 K604R knock-in HCT116^+/+^ cell lines were generated using CRISPR/Cas9 mediated-mutagenesis. sgRNA-pX458 and repair template were co-transfected into HCT116^+/+^ cells using Lipo8000 Transfection Reagent, and 36 h after transfection, GFP-positive single cells were sorted by FACS into the 96-well plates. Cells were cultured for 14–21 days in cell incubator. When clones reached 60%–70% confluency, genomic DNA was extracted for PCR verification of successful knock-in. sgRNA sequences are provided in Table S1.

### Immunoprecipitation

Cells were lysed in lysis buffer at 4°C for 30 min. Lysates were centrifuged at 12,000 rpm for 15 min at 4°C. Supernatants were collected and incubated with appropriate antibody-conjugated beads at 4°C for 4 h. Immunoprecipitated proteins were washed 3 times with lysis buffer and analyzed by immunoblotting.

### Dot blot

Targeted peptides were diluted in ddH_2_O with appropriate ratio. Samples were loaded on NC membranes and air-dried for 10 min at room temperature. The membranes were blocked with 5% fat-free milk (BD Biosciences) and probed with glutaminylation antibody.

### Ubiquitination assay

Cells were co-transfected with Flag/Myc-tagged plasmids along with HA-tagged ubiquitin. MG132 treatment or glutamine supplementation was administered 36 h posttransfection. Cells were collected and lysed in ubiquitination lysis buffer (1% SDS, 100 mmol/L Tris-HCl pH 7.5, 0.5 mmol/L EDTA, 1 mmol/L DTT), followed by boiling at 95°C for 10 min. The lysates were diluted 10-fold in regular lysis buffer, followed by immunoprecipitation and immunoblotting.

### *In vitro* pull-down assay

Flag- and HA-tagged proteins purified from transfected HCT116^+/+^ cells using immunoprecipitation. HA-tagged protein was eluted from HA-beads and combined with HA-peptide. HA-peptide and Flag-beads were subjected to immunoprecipitation and results were analyzed by immunoblotting.

### EdU staining assay

Cultured cells were incubated with 20 μmol/L EdU (final concentration) for 1.5 h. Cells were washed 3 times with PBS and fixed with 4% paraformaldehyde for 20 min at room temperature. Permeabilization was performed using 0.5% Triton X-100 in PBS for 10 min. Cells were then incubated with Click-iT working solution (YF488 Click-iT EdU kit, Beijing, China) in dark for 30 min. Nuclei were stained with DAPI or Hoechst 33342 and results were analyzed by fluorescence microscope or Flow cytometry.

### Identification of FBW7 glutaminylation site

HCT116^+/+^ cells were transfected with pcDNA3.1-FBW7-Flag or co-transfected with pcDNA3.1-FBW7-Flag and pcDNA3.1-QARS-Flag. After 36 h, cells were treated with different concentrations of glutamine, followed by immunoprecipitation. Samples were then washed 3 times with 50-mmol/L NH_4_HCO_3_ (pH 8.0) and digested with trypsin overnight. An additional 3-hour trypsin digestion was performed prior to boiling the samples at 99°C. The supernatant was collected by centrifugation, vacuum-dried, and subjected to UPLC-QE MS/MS analysis.

The relative abundance of glutaminylated peptides was quantified using the equation: intensity of Gln-K peptide/(intensity of Gln-K peptide + intensity of non-Gln-K peptide) = Ratio of Gln-K. This method was performed according to a published method.

### Tandem affinity purification (TAP)

HCT116^+/+^ cells were transfected with pcDNA3.1-Flag or pcDNA3.1-QARS-Flag. After 36 h, immunoprecipitation was performed. To confirm transfection efficiency, samples were subjected to SDS-PAGE. Samples were then washed 3 times with 50-mmol/L NH_4_HCO_3_ (pH 8.0) and digested with trypsin for 3 h. The resulting peptides were collected by centrifugation, vacuum-dried, and analyzed by ultrahigh-performance liquid chromatography coupled with Q-Exactive MS/MS (UPLC-QE MS/MS) detection.

### *In vitro* glutaminylation reaction

*In vitro* glutaminylation reactions were performed in a 30-μL reaction system containing: 50-mmol/L HEPES pH 7.5, 25-mmol/L KCl, 2-mmol/L MgCl_2_, 2-mmol/L glutamine, 4-mmol/L ATP, 10-nmol/L QARS, 0.05-μg/μL synthetic substrate peptide, or recombinant FBW7 protein. Before adding QARS, the reaction pH was adjusted to 7.5. The reaction was carried out at 37°C for 3 h. Samples were desalted and analyzed by MALDI-TOF/TOF mass spectrometer (MALDI-TOF/TOF-MS) (SCIEeX-5800). FBW7 protein modifications were further validated by immunoblotting.

### *In vitro* de-glutaminylation reaction

*In vitro* de-glutaminylation was performed in a 30-μL reaction system containing: 50-mmol/L HEPES pH 7.5, 6-mmol/L MgCl_2_, 1-mmol/L DTT, 1-mmol/L NAD^+^, 0.05-μg/μL synthetic glutaminylation peptide, 1-mg/mL SIRTs, and 1-mmol/L PMSF. Reactions were incubated at 37°C for 4 h. Peptide modifications were analyzed using MALDI-TOF/TOF-MS (SCIEeX-5800).

### Metabolites measurement by LC–MS/MS

Cultured cells were treated with iced methanol (Sigma Aldrich, Saint Louis, USA): ddH_2_O = 4:1 (*v*/*v*) and quickly harvested for metabolite extraction at −80°C overnight. The samples were centrifuged at 12,000 rpm for 15 min at 4°C, and the supernatant was collected for analysis using AB SCIEX TripleTOF 6600 + LC-MS/MS.

### Structural analysis (protein–protein docking)

Glutamine modification at the K604 site of FBW7^WT^ was performed using Alphafold generated structures and Schrodinger software. Due to limitations in ZDOCK software performance, protein–protein docking of full-length FBW7 with Mcl-1 or c-Myc could not be completed because of the excessively large molecular weight. Therefore, the first 310 amino acids of FBW7 (non-WD40 recognition region) were removed, and the first 30 amino acids of c-Myc (non-phosphorylated region) were also excluded. When docking FBW7^WT^ with Mcl-1 or c-Myc, the parameter settings for “active amino acids” were set as position 462, 465, 479, 505, and 519. For docking FBW7^Gln-K604^ with Mcl-1 or c-Myc, the active amino acids for FBW7^Gln-K604^ were set as position 604.

### Statistical analysis

All statistical analyses were performed using GraphPad Prism 9. Data are presented as mean ± SEM. Statistical significance was determined using: unpaired 2-tailed Student’s *t*-test and 2-way ANOVA. Each data point represents an individual measurement. Statistical significance was defined as: ns denotes *P*-values > 0.05, *P < 0.05, **P < 0.01, ***P < 0.001.

Western blots and cell cycle content measurements were performed in at least 3 independent experiments. Microscopy images were randomly selected from 5 or more regions of interest, with representative images shown.

## Supplementary data

Supplementary data is available at *Protein & Cell* online https://doi.org/10.1093/procel/pwaf029.

pwaf029_Supplementary_Figures_S1-S7

pwaf029_Supplementary_Table_S1

## Data Availability

The data generated in this study are available within the article and its supplementary data files.
