# Supplementary material for: Glutamine signaling specifically activates c-Myc and Mcl-1 to facilitate cancer cell proliferation and survival
Source: Protein Cell. 2025 May 4;16(11):968–84. doi: 10.1093/procel/pwaf029 (PMC12698188; doi:10.1093/procel/pwaf029)
Supplement: pwaf029_Supplementary_Figures_S1-S7 [file pwaf029_supplementary_figures_s1-s7.pdf]

Fig. S1 QARS glutaminylates FBW7 K604 (related to Fig. 2). (A), Co-immunoprecipitation showing interactions between ectopically expressed c-Myc and QARS. (B), Ubiquitination levels of ectopically expressed c-Myc in HCT116$^{+/+}$, *FBW7*-silenced HCT116$^{+/+}$ cells with or without 4 mmol/L glutamine. (C), MS/MS spectra confirming the identification of FBW7 Gln-K604 (upper) compared with synthetic Gln-K604 peptide (lower). (D and E), Characterization of site-specific Gln-K604 antibody. Antibody specificity tested against Gln-K604-containing and non-glutaminylated peptides. Peptide sequences are shown below (D). Reactivity of the antibody with wild-type FBW7, FBW7 K604R, and FBW7 K604Q mutants (E). (F), Intracellular glutamine levels in glutamine-starved HCT116$^{+/+}$ cells transferred to glutamine-free or 4 mmol/L glutamine-containing media for 2 h (n=3).

Fig. S2 Gln-K604 specifically induces c-Myc and Mcl-1 (related to Fig. 3). (A), Endogenous Mcl-1 levels in untreated HCT116$^{+/+}$ cells and cells treated with methyl-α-ketoglutarate, glutamate, or glutamine. (B), Effects of glutamine supplementation on other cell cycle regulators levels in HCT116$^{+/+}$ cells. (C), DNA sequencing confirming successful generation of K604Q KI and K604R KI cells, using HCT116$^{+/+}$ cells as a reference. (D), Levels of Rb, Notch1, Jun, Cyclin E1 and Cdh1 in HCT116$^{+/+}$, K604Q KI and K604R KI cells.

Fig. S3 Gln-K604 disrupts the binding of c-Myc and Mcl-1 to FBW7 (related to Fig. 4). (A-C), Ubiquitination levels of ectopically expressed Cyclin E1 (A), Notch (B), and Jun (C) in glutamine-starved HCT116$^{+/+}$ and K604Q KI cells with or without 4 mmol/L glutamine. (D), Schematic representation of FBW7 (upper) and its Δ6th WD40 deletion mutant FBW7$^{\Delta 6thWD40}$ (lower). (E-G), Ubiquitination levels of ectopically expressed Cyclin E1 (E), Notch (F) and Jun (G) in HCT116$^{+/+}$, FBW7-overexpressing HCT116$^{+/+}$, and FBW7Δ^{6thWD40}-overexpressing HCT116$^{+/+}$ cells. (H), Co-immunoprecipitation showing the effects of glutamine supplementation on FBW7-c-Myc and FBW7-Mcl-1 interactions in HCT116$^{+/+}$ cells. (I), Structural analysis of interaction between FBW7^{WT}/FBW7$^{Gln\text{-}K604}$ and c-Myc (upper) and interaction between FBW7^{WT}/FBW7$^{Gln\text{-}K604}$ and Mcl-1 (lower). Close-up view of the FBW7-c-Myc/Mcl-1 interacting amino acids are shown on the right. (J and K), Binding of c-Myc and Mcl-1 to FBW7 in HCT116$^{+/+}$ and K604R KI cells under 0 mmol/L or 4 mmol/L glutamine (J) or with QARS overexpression (K).

Fig. S4 Cell cycle-dependent Gln-K604 promotes anabolism and apoptosis resistance (related to Fig. 5). (A), EdU incorporation into DNA of HCT116$^{+/+}$, K604Q KI, and K604R KI cells, analyzed via fluorescence-activated cell sorting. (B), Cell cycle distribution of ALS-arrested HCT116$^{+/+}$ and K604R KI cells in the presence or absence of 4 mmol/L glutamine, quantified by flow cytometry (n=3). (C and D), Apoptotic rate of ALS-treated HCT116$^{+/+}$ and K604R KI cells with QARS overexpression (C) or glutamine supplementation (D). (E and F), Effects of *QARS* silencing on apoptosis of ALS-(E) and HU-treated (F) HCT116$^{+/+}$ and K604R KI cells. (G and H), Apoptotic rates of ALS-treated and *MCL-1*-silenced HCT116$^{+/+}$ cells with QARS overexpression (G) or glutamine supplementation (H), assayed by FCM.

Fig. S5 SIRT1 removed Gln-K604 and reversed Gln-K604 effects in vitro (related to Fig. 6). (A and B), Cell cycle distribution of untreated or HU-treated (A) and ALS-treated (B) HCT116$^{+/+}$, *SIRT1*-silenced HCT116$^{+/+}$, K604R KI and *SIRT1*-silenced K604R KI cells, assayed by FCM (n=3).

Fig. S6 Precise chemotherapy-sensitizing strategies for Gln-K604-intact and Gln-K604-null cancers (related to Fig. 7). (A), Overview of cancer types with different FBW7 and Gln-K604 statuses. (B and C), Responses of FBW7 substrates to glutamine starvation (B) and *QARS* silencing (C) in Gln-K604-intact and Gln-K604-null cells. (D), Apoptotic rates in Gln-K604-intact and Gln-K604-null cells with glutaminol, ALS, or both.

Fig. S7 FBW7 glutamine signaling (related to the Discussion). Schematic representation of the FBW7 signaling network. QARS glutaminylates FBW7 K604 to form Gln-K604, which specifically activates c-Myc to promote glutamine uptake and anabolism. Gln-K604 also enhances Mcl-1 activity, conferring apoptosis resistance. SIRT1 reverses Gln-K604 and its effects.

Fig. S1

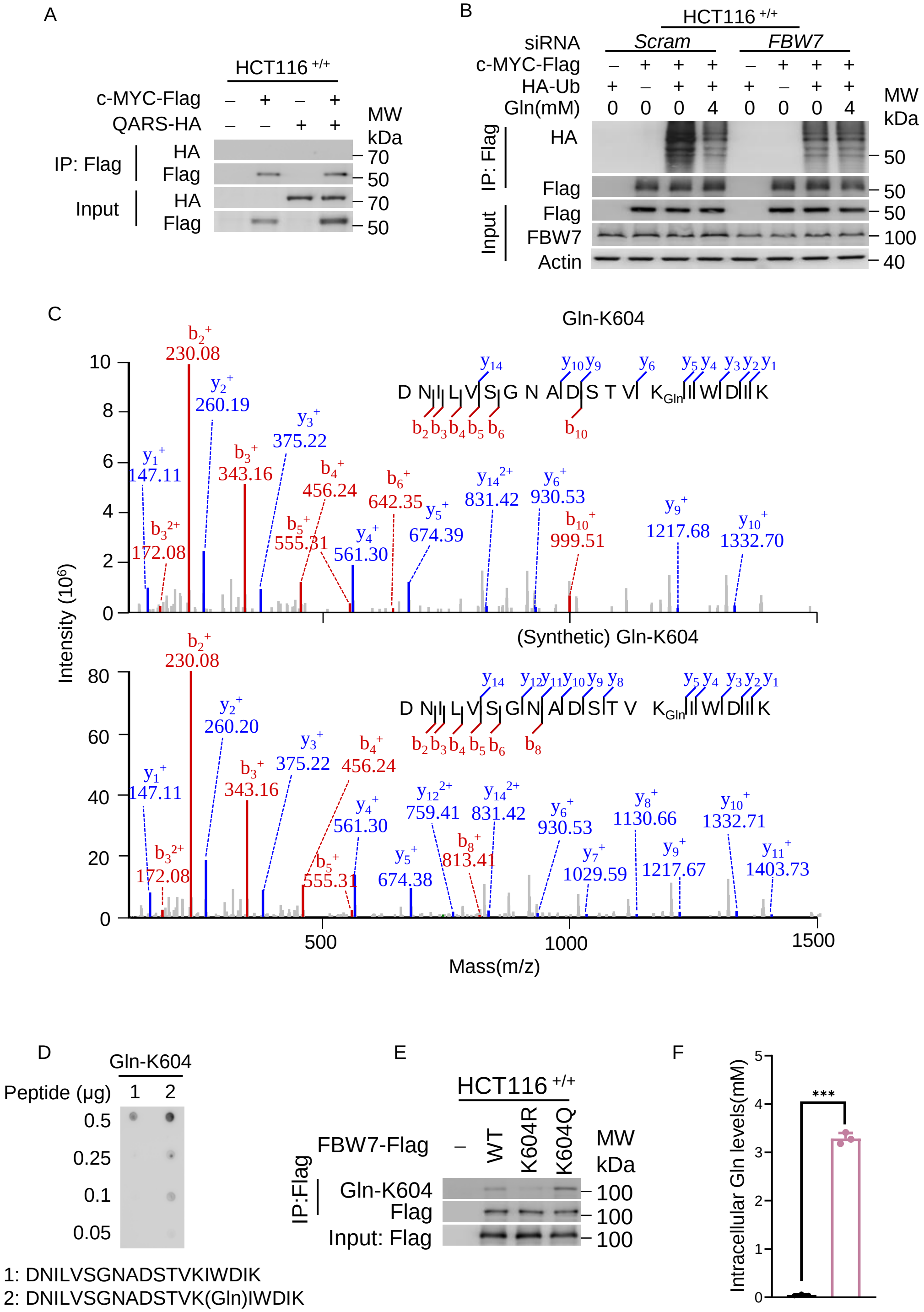
A
HCT116 +/+
c-MYC-Flag – + – +
QARS-HA – – + +
IP: Flag
Input
HA
Flag
MW kDa
70
50
B
HCT116 +/+
siRNA
Scram
FBW7
c-MYC-Flag – + + + – + + +
HA-Ub + – + + + – + +
Gln(mM) 0 0 0 4 0 0 0 4
IP: Flag
Input
HA
Flag
FBW7
Actin
MW kDa
50
100
40
C
Gln-K604
(Synthetic) Gln-K604
Intensity (10^6)
Mass(m/z)
D N I L V S G N A D S T V K Gln I W D I K
b2+ 230.08
y2+ 260.19
y3+ 375.22
y1+ 147.11
b3+ 343.16
b4+ 456.24
b6+ 642.35
y5+ 674.39
y14 2+ 831.42
y6+ 930.53
b3 2+ 172.08
b5+ 555.31
y4+ 561.30
b10+ 999.51
y9+ 1217.68
y10+ 1332.70
b2+ 230.08
y2+ 260.20
y3+ 375.22
b4+ 456.24
y1+ 147.11
b3+ 343.16
y4+ 561.30
y12 2+ 759.41
y14 2+ 831.42
y6+ 930.53
y8+ 1130.66
y10+ 1332.71
b3 2+ 172.08
b5+ 555.31
y5+ 674.38
b8+ 813.41
y7+ 1029.59
y9+ 1217.67
y11+ 1403.73
500
1000
1500
D
Gln-K604
Peptide (μg) 1 2
0.5
0.25
0.1
0.05
1: DNILVSGNADSTVKIWDIK
2: DNILVSGNADSTVK(Gln)IWDIK
E
HCT116 +/+
FBW7-Flag – WT K604R K604Q
IP:Flag
Gln-K604
Flag
Input: Flag
MW kDa
100
F
Intracellular Gln levels(mM)
***
0
1
2
3
4
5

Fig. S2

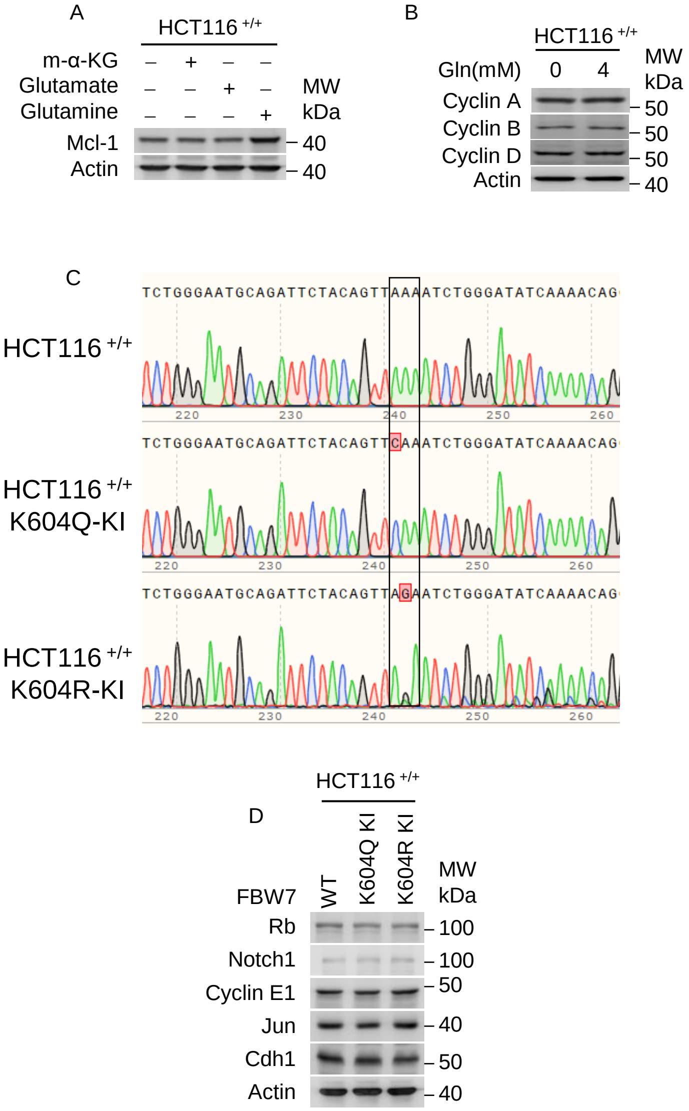
A
HCT116 +/+
m-α-KG – + – –
Glutamate – – + –
Glutamine – – – +
MW kDa
Mcl-1 – 40
Actin – 40
B
HCT116 +/+
Gln(mM) 0 4
MW kDa
Cyclin A – 50
Cyclin B – 50
Cyclin D – 50
Actin – 40
C
TCTGGGAATGCAGATTCTACAGTTAAAATCTGGGATATCAAAACAG
HCT116 +/+
220 230 240 250 260
TCTGGGAATGCAGATTCTACAGTTCAAATCTGGGATATCAAAACAG
HCT116 +/+ K604Q-KI
220 230 240 250 260
TCTGGGAATGCAGATTCTACAGTTAGAATCTGGGATATCAAAACAG
HCT116 +/+ K604R-KI
220 230 240 250 260
D
HCT116 +/+
FBW7
WT
K604Q KI
K604R KI
MW kDa
Rb – 100
Notch1 – 100
Cyclin E1 – 50
Jun – 40
Cdh1 – 50
Actin – 40

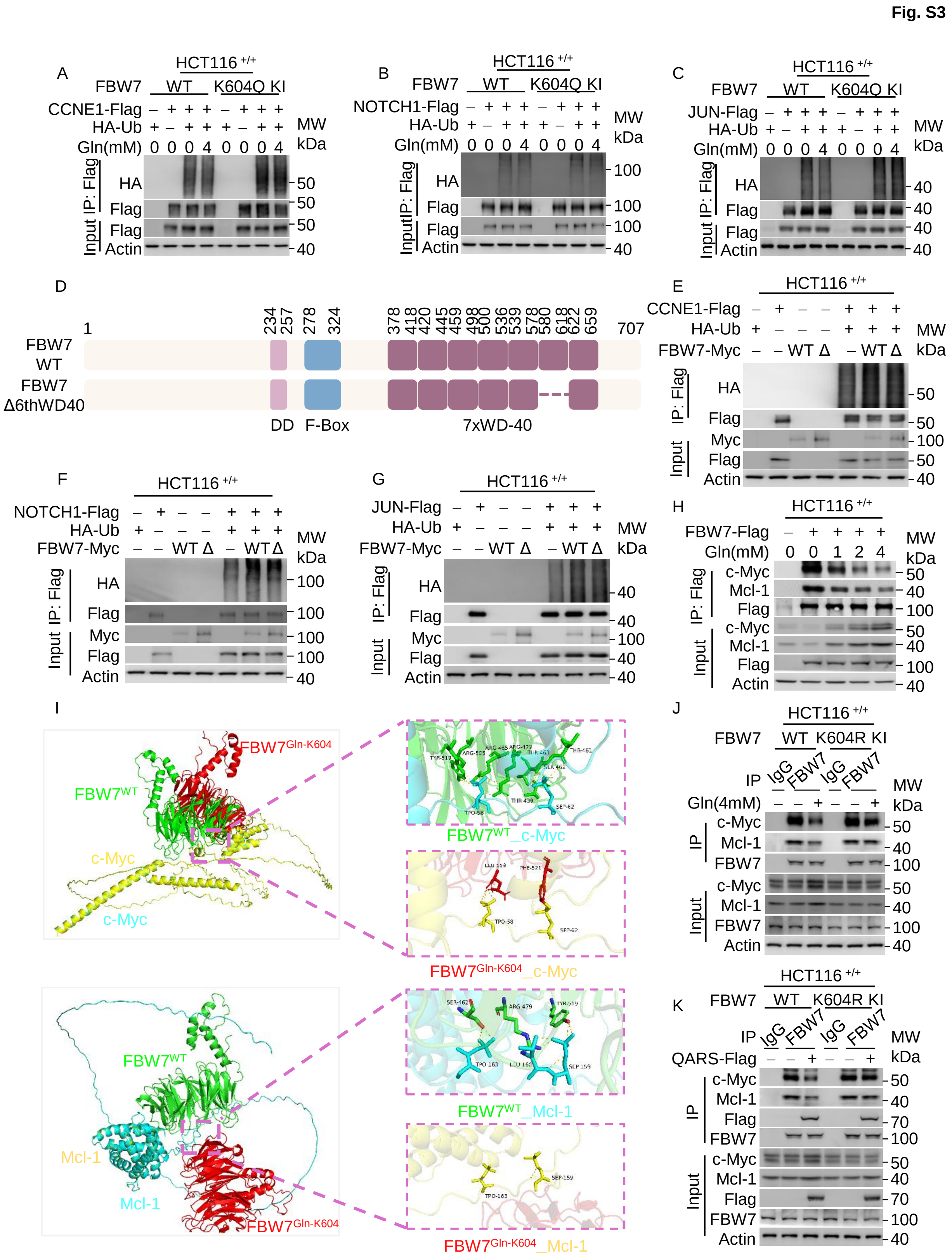
Fig. S3
A
HCT116 +/+
FBW7 WT K604Q KI
CCNE1-Flag
HA-Ub
Gln(mM) 0 0 0 4 0 0 0 4
MW kDa
IP: Flag
HA
Flag
Input
Flag
Actin
B
NOTCH1-Flag
C
JUN-Flag
D
FBW7 WT
FBW7 Δ6thWD40
DD
F-Box
7xWD-40
E
FBW7-Myc
Myc
F
G
H
FBW7-Flag
Gln(mM) 0 0 1 2 4
c-Myc
Mcl-1
I
FBW7Gln-K604
FBW7WT
FBW7WT_c-Myc
FBW7Gln-K604_c-Myc
FBW7WT_Mcl-1
FBW7Gln-K604_Mcl-1
J
FBW7 WT K604R KI
IP IgG FBW7 IgG FBW7
Gln(4mM)
K
QARS-Flag

Fig. S4

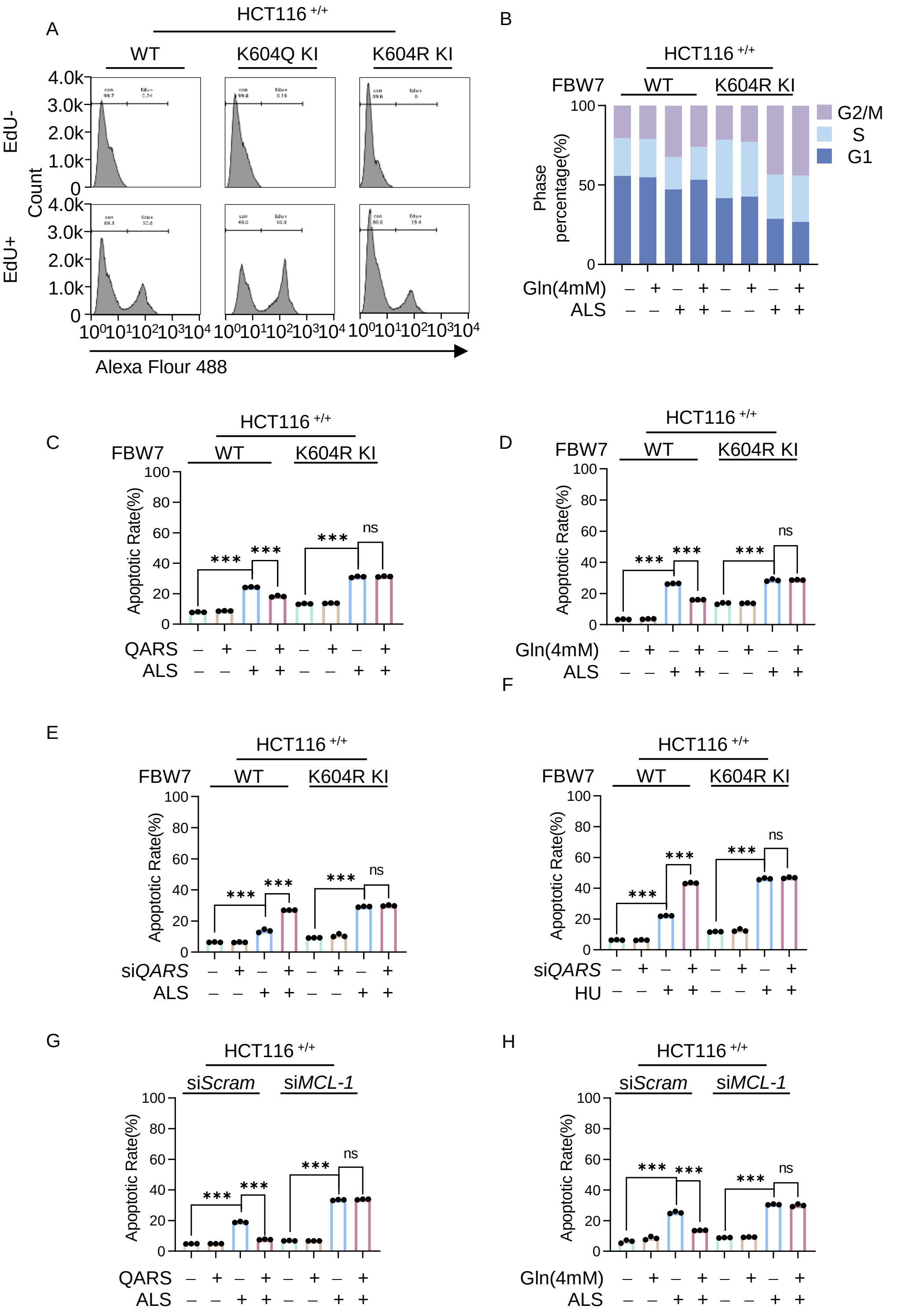
A
HCT116 +/+
WT
K604Q KI
K604R KI
EdU-
EdU+
Count
Alexa Flour 488
B
HCT116 +/+
FBW7
WT
K604R KI
Phase percentage(%)
G2/M
S
G1
Gln(4mM)
ALS
C
HCT116 +/+
FBW7
WT
K604R KI
Apoptotic Rate(%)
QARS
ALS
***
ns
D
HCT116 +/+
FBW7
WT
K604R KI
Apoptotic Rate(%)
Gln(4mM)
ALS
E
HCT116 +/+
FBW7
WT
K604R KI
Apoptotic Rate(%)
siQARS
ALS
F
HCT116 +/+
FBW7
WT
K604R KI
Apoptotic Rate(%)
siQARS
HU
G
HCT116 +/+
siScram
siMCL-1
Apoptotic Rate(%)
QARS
ALS
H
HCT116 +/+
siScram
siMCL-1
Apoptotic Rate(%)
Gln(4mM)
ALS

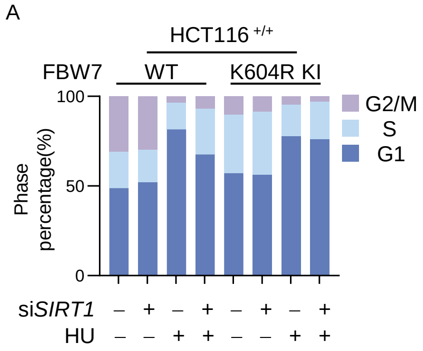
A
HCT116 +/+
FBW7
WT
K604R KI
100
50
0
Phase
percentage(%)
G2/M
S
G1
siSIRT1 – + – + – + – +
HU – – + + – – + +

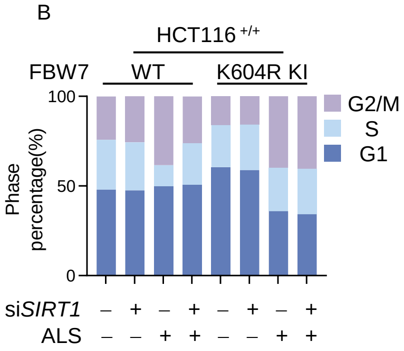
B
HCT116 +/+
FBW7
WT
K604R KI
100
50
0
Phase
percentage(%)
G2/M
S
G1
siSIRT1 – + – + – + – +
ALS – – + + – – + +

Fig. S6

A

FBW7	Cell Line	Variant Type	Protein Change	Tumor Type
WT	HCT116$^{+/+}$	WT	No Change	Colorectal Cancer
	HCT116$^{-/-}$	WT	No Change	Colorectal Cancer
	SW480	WT	No Change	Colorectal Cancer
	SW620	WT	No Change	Colorectal Cancer
	OV-3	WT	No Change	Ovarian Cancer
	UMUC3	WT	No Change	Bladder Cancer
Mutation	SW837	INS	p.L403fs	Colorectal Cancer
	SNUC4	INS	p.R473fs	Colorectal Cancer
	SNU1040	DEL	p.T166fs	Colorectal Cancer
	SW48	DEL	p.G667fs	Colorectal Cancer
	22RV1	DEL	p.E117del	Prostatic Cancer
	SK-OV-3	SNP	p.R505L	Ovarian Cancer
	LOVO	SNP	p.R505C	Colorectal Cancer
	SW1116	SNP	p.H460Y	Colorectal Cancer

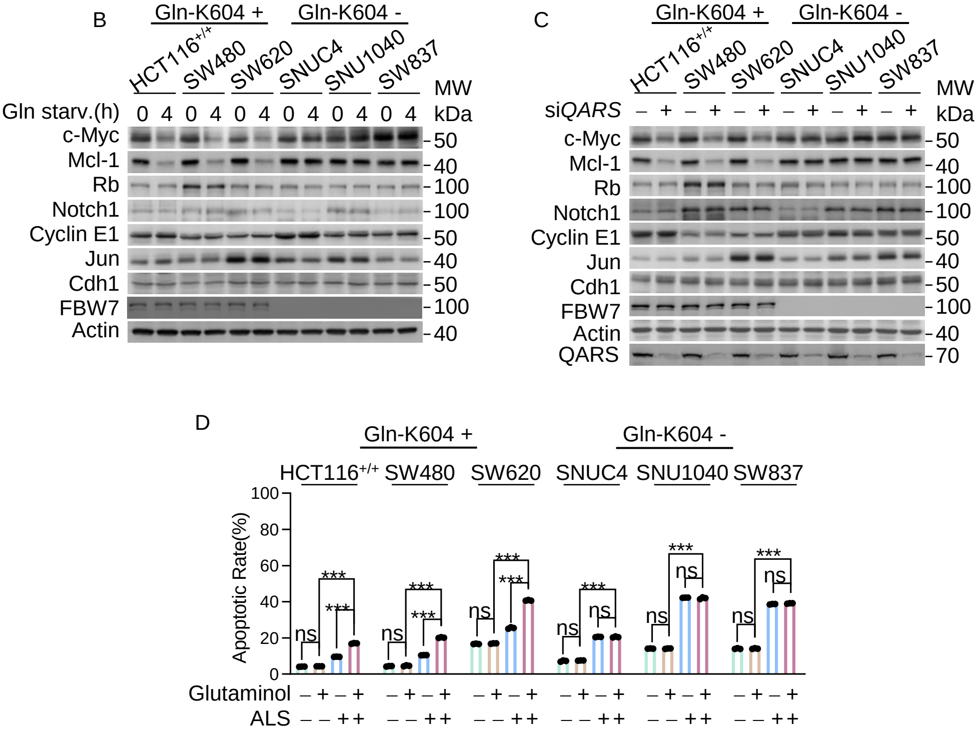

Fig. S7

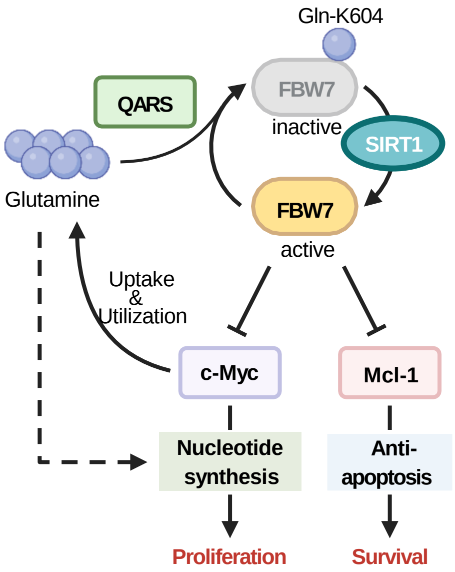
Gln-K604
FBW7
inactive
QARS
SIRT1
Glutamine
FBW7
active
Uptake
&
Utilization
c-Myc
Mcl-1
Nucleotide synthesis
Anti-apoptosis
Proliferation
Survival
