# Supplementary material for: Glutamine signaling specifically activates c-Myc and Mcl-1 to facilitate cancer cell proliferation and survival
Source: Protein Cell. 2025 May 4;16(11):968–84. doi: 10.1093/procel/pwaf029 (PMC12698188; doi:10.1093/procel/pwaf029)
Supplement: pwaf029_Supplementary_Table_S1 [file pwaf029_supplementary_table_s1.docx]

| Oligonucleotides | | |
| --- | --- | --- |
| PCR FBW7-F: AACGGGCCCTCTAGACTCGAGATGAATCAGGAACTG CTCTCTGTG | This Study | N/A |
| PCR FBW7-R: TAGTCCAGTGTGGTGGAATTCCTTCATGTCCACATCAAAGTCCAG | This Study | N/A |
| PCR FBW7-K604R-F: ACAGTTAAGATCTGGGATATCAAAACAGGACAGTG | This Study | N/A |
| PCR FBW7-K604R-R: TCCCAGATCTTAACTGTAGAATCTGCATTCCCAGA | This Study | N/A |
| PCR FBW7^Δ6thWD40^-F: CAAAGCATCAGAGTGCTGTGACCTGTTTACAGT | This Study | N/A |
| PCR FBW7^Δ6thWD40^-R: CAGCACTCTGATGCTTTGTTAACGTGTGAATGCAATTCC | This Study | N/A |
| PCR QARS-F: AACGGGCCCTCTAGACTCGAGATGGCGGCTCTAGACTCCCT | This Study | N/A |
| PCR QARS-R: TAGTCCAGTGTGGTGGAATTCCACCTTTCCTGGGTCTTCCTTC | This Study | N/A |
| PCR c-MYC-F: AACGGGCCCTCTAGACTCGAGCTGGATTTTTTTCGGGTAGTG | This Study | N/A |
| PCR c-MYC-R: TAGTCCAGTGTGGTGGAATTCCGCACAAGAGTTCCGTAGCTG | This Study | N/A |
| PCR MCL-1-F: AACGGGCCCTCTAGACTCGAGATGTTTGGCCTCAAAAGAAACG | This Study | N/A |
| PCR MCL-1-R: TAGTCCAGTGTGGTGGAATTCTCTTATTAGATATGCCAAACCAGCTC | This Study | N/A |
| PCR CCNE1-F: AACGGGCCCTCTAGACTCGAGATGCCGAGGGAGCGCAGG | This Study | N/A |
| PCR CCNE1-R: TAGTCCAGTGTGGTGGAATTCCGCCATTTCCGGCCCGCT | This Study | N/A |
| PCR JUN-F: AACGGGCCCTCTAGACTCGAGATGACTGCAAAGATGGAAACGAC | This Study | N/A |
| PCR JUN-R: TAGTCCAGTGTGGTGGAATTCAAATGTTTGCAACTGCTGCGT | This Study | N/A |
| PCR NOTCH1-F: AACGGGCCCTCTAGACTCGAGGTGCTGCTGTCCCGCAAG | This Study | N/A |
| PCR NOTCH1-R: TAGTCCAGTGTGGTGGAATTCCTTGAAGGCCTCCGGAATG | This Study | N/A |
| PCR SIRT1-F: AACGGGCCCTCTAGACTCGAGATGGCGGACGAGGCGGCC | This Study | N/A |
| PCR SIRT1-R: TAGTCCAGTGTGGTGGAATTCTGATTTGTTTGATGGATAG | This Study | N/A |
| PCR SIRT2-F: AACGGGCCCTCTAGACTCGAGATGGACTTCCTGCGGAACT | This Study | N/A |
| PCR SIRT2-R: TAGTCCAGTGTGGTGGAATTCCTGGGGTTTCTCCCTCTCT | This Study | N/A |
| PCR SIRT6-F: AACGGGCCCTCTAGACTCGAGATGTCGGTGAATTACGCGG | This Study | N/A |
| PCR SIRT6-R: TAGTCCAGTGTGGTGGAATTCGCTGGGGACCGCCTTGGCC | This Study | N/A |
| PCR SIRT7-F: AACGGGCCCTCTAGACTCGAGATGGCAGCCGGGGGTCTG | This Study | N/A |
| PCR SIRT7-R: TAGTCCAGTGTGGTGGAATTCCGTCACTTTCTTCCTTTTTGT | This Study | N/A |
| siRNA FBW7 (human): AACCUUCUCUGGAGAGAGAAAUGTT | This Study | N/A |
| siRNA QARS (human): CAAAGGAUGUGGUGGAGAATT | This Study | N/A |
| siRNA c-MYC (human): CGGUGCAGCCGUAUUUCUATT | This Study | N/A |
| siRNA MCL-1 (human): CGCCGAAUUCAUUAAUUUATT | This Study | N/A |
| CRISPR/Cas9-mediated FBW7 gene editing primer-F: CACCGCAGATTCTACAGTTAAAATC | This Study | N/A |
| CRISPR/Cas9-mediated FBW7 gene editing primer-R: AAACGATTTTAACTGTAGAATCTGC | This Study | N/A |
| QPCR-SLC1-F: TCATGTGGTACGCCCCTGT | This Study | N/A |
| QPCR-SLC1-R: GCGGGCAAAGAGTAAACCCA | This Study | N/A |
| QPCR-ACTIN-F: CATGTACGTTGCTATCCAGGC | This Study | N/A |
| QPCR-ACTIN-R: CTCCTTAATGTCACGCACGAT | This Study | N/A |
